# Nintedanib induces senolytic effect via STAT3 inhibition

**DOI:** 10.1038/s41419-022-05207-8

**Published:** 2022-09-02

**Authors:** Hyun-Ji Cho, Jeong-A Hwang, Eun Jae Yang, Eok-Cheon Kim, Jae-Ryong Kim, Sung Young Kim, Young Zoon Kim, Sang Chul Park, Young-Sam Lee

**Affiliations:** 1 Department of New Biology, DGIST, Daegu, 42988 Korea; 2 Well Aging Research Center, DGIST, Daegu, 42988 Korea; 3 Department of Biochemistry and Molecular Biology, Smart-Aging Convergence Research Center, College of Medicine, Yeungnam University, Daegu, 42415 Korea; 4 Department of Biochemistry, Konkuk University School of Medicine, Seoul, 05029 Korea; 5 Division of Neuro-Oncology and Department of Neurosurgery, Samsung Changwon Hospital, Sungkyunkwan University School of Medicine, Changwon, 51353 Korea; 6 The Future life and Society Research Center, Chonnam National University, Gwangju, 58128 Korea; 7 New Biology Research Center, DGIST, Daegu, 42988 Korea

**Keywords:** Senescence, Apoptosis

## Abstract

Selective removal of senescent cells, or senolytic therapy, has been proposed to be a potent strategy for overcoming age-related diseases and even for reversing aging. We found that nintedanib, a tyrosine kinase inhibitor, selectively induced the death of primary human dermal fibroblasts undergoing RS. Similar to ABT263, a well-known senolytic agent, nintedanib triggered intrinsic apoptosis in senescent cells. Additionally, at the concentration producing the senolytic effect, nintedanib arrested the cell cycle of nonsenescent cells in the G1 phase without inducing cytotoxicity. Interestingly, the mechanism by which nintedanib activated caspase-9 in the intrinsic apoptotic pathway differed from that of ABT263 apoptosis induction; specifically, nintedanib did not decrease the levels of Bcl-2 family proteins in senescent cells. Moreover, nintedanib suppressed the activation of the JAK2/STAT3 pathway, which caused the drug-induced death of senescent cells. STAT3 knockdown in senescent cells induced caspase activation. Moreover, nintedanib reduced the number of senescence-associated β-galactosidase-positive senescent cells in parallel with a reduction in STAT3 phosphorylation and ameliorated collagen deposition in a mouse model of bleomycin-induced lung fibrosis. Consistently, nintedanib exhibited a senolytic effect through bleomycin-induced senescence of human pulmonary fibroblasts. Overall, we found that nintedanib can be used as a new senolytic agent and that inhibiting STAT3 may be an approach for inducing the selective death of senescent cells. Our findings pave the way for expanding the senolytic toolkit for use in various aging statuses and age-related diseases.

## Introduction

Cellular senescence is a crucial biological process underlying aging [1] and is caused by various factors, including genomic instability, oxidative damage, telomere attrition and dysfunction [2]. Due to the detrimental effects of senescent cells in aged tissues, including impaired regeneration caused by decreased proliferative capacity and increased chronic inflammation caused by the enhanced senescence-associated secretory phenotypes (SASPs), developing senotherapeutics that selectively target cells undergoing senescence may lead to tissue homeostasis and advance the pursuit of healthy aging and longevity while overcoming age-related diseases. Notably, the term “aging-related” (XT9T) has been updated in the extension code within the International classification of disease (ICD-11) of the World Health Organization. Aging-related in the ICD-11 is now described to be “caused by biological processes which persistently lead to the loss of organism’s adaptation and progress in older ages” [3, 4].

Senotherapeutics are both senomorphics, which restore cellular functions in senescent cells, and senolytics, which selectively eliminate senescent cells [5, 6]. In particular, since proof-of-concept evidence for a senolytic strategy has been reported, such as through the elimination of p16^Ink4a^-positive senescent cells to inhibit tissue dysfunction, improve the health span and extend the lifespan [7–11], several senolytic agents have been identified. Typically, senolytic agent modes of action inhibit pro-survival pathways or cell survival regulatory factors. Most confirmed senolytics attenuate antiapoptotic features in senescent cells. For example, the dasatinib and quercetin (D + Q) combination inhibits the activity cell survival regulatory factors such as Bcl-xL, PAI-1 and p21 to selectively eliminate senescent cells [11]. ABT263 (also known as navitoclax) targets anti-apoptotic proteins Bcl-2 and Bcl-xL [9, 12]. In particular, the first clinical trial with the D + Q combination confirmed that this cotreatment attenuated physical dysfunction in patients with idiopathic pulmonary fibrosis (IPF) [13]. Recently, bis-2-(5-phenylacetamido-1,3,4-thiadiazol-2-yl)ethyl sulfide (BPTES), which is an inhibitor of glutaminase 1 [14], azithromycin and roxithromycin, which are known antibiotics [15], and R406, which is a known spleen tyrosine kinase (SYK) inhibitor [16], were reported to constitute a new family of senolytic drugs.

Nintedanib is a tyrosine kinase inhibitor that exerts antiangiogenic activity via blockade of endothelial growth factor receptor (EGFR), fibroblast growth factor receptor (FGFR), and platelet-derived growth factor receptor (PDGFR) [17]. It was originally developed as an anticancer agent [18]. Recent results have shown that nintedanib induces apoptosis in triple-negative breast cancer cells by inhibiting the phosphorylation of STAT3 [19] and inhibits the tumor growth of malignant pleural mesothelioma (MPM) via blockade of angiogenesis [20]. Nintedanib in combination with docetaxel has been used for the treatment of lung cancer [21]. Moreover, this agent exerts an antifibrotic effect that reduces the rate of pulmonary fibrosis progression [22] and is one of two US Food and Drug Administration (FDA)-approved drugs (the other is pirfenidone) for the treatment of IPF [23].

In our study, among the senolytic candidates identified from previously reported high-throughput screening results [16], nintedanib selectively reduced the viability of senescent human dermal fibroblasts (HDFs) and reduced the number of senescent cells involved in a mouse model of bleomycin-induced lung fibrosis. We determined that the nintedanib-induced senolytic effect was mediated via STAT3 inhibition, indicating that this agent is a potential senolytic with a mode of action that differs from that of other known senolytic agents.

## Materials and methods

### Cells and culture conditions

Human dermal fibroblasts (HDFs) were purchased from ATCC (PCS-201-010, Manassas, VA, USA). Cells were cultured in growth medium composed of DMEM (Welgene, Korea) supplemented with 10% FBS (Welgene) and antibiotic-antimycotic solution (100 units/ml penicillin, 100 ug/ml streptomycin and 250 ng/ml amphotericin B, Welgene) at 37 °C in an incubator with 5% CO_2_. We used senescent cells, defined as those for which the population doubling time (DT) was more than 14 days, and nonsenescent cells, defined as those for which the DT was less than one day [24]. Human pulmonary fibroblasts (HPFs) were purchased from Promocell (C-12360, Heidelberg, Germany). The cells were cultured in growth medium composed of Fibroblast Growth Medium 2 (FGM-2, Promocell, Germany) supplemented with SupplementMix (Promocell) containing 2% FCS, 1 ng/ml basic fibroblast growth factor and 5 μg/ml insulin at 37 °C in an incubator with 5% CO_2_. To induce bleomycin-induced cellular senescence in human pulmonary fibroblasts (HPFs), we treated with bleomycin (25, 50, and 75 μg/ml) for five days. The levels of bleomycin-induced senescence were investigated western blot using anti-p16 and -p21 antibodies. Mycoplasma and microbial contamination were checked monthly and cell doubling time and morphological change were routinely checked.

### Drug preparation

Nintedanib (S1010), WP1066 (S2796) and ABT263 (S1001) were purchased from Selleckchem (Houston, TX, USA). Stock solutions (10 mM) were prepared in DMSO. They were added to culture medium to generate a suitable working solution. As a vehicle control, DMSO at the same volume was added to culture medium. Bleomycin sulfate (B5507) was purchased from Sigma-Aldrich (St. Louis, MO). Stock solution (3 U/ml) was prepared in sterile water.

### Cell viability and cytotoxicity assays

Cell viability and proliferation were evaluated using a water-soluble tetrazolium salt-based D-plus cell viability and cytotoxicity assay reagent (CCK-8 reagent, Dongin LS, Korea). Cells were seeded at 1 × 10^4^ cells/well in 96-well plates and treated with 1, 2, 5, 10 or 20 μM nintedanib in the growth medium for 1, 2, or 3 days. The cells were then treated with 10 μl of CCK-8 reagent and incubated for 4 h. After the incubation, the absorbance of the wells was read at 450 nm using a microplate reader (Infinite M200PRO, TECAN Trading AG, Switzerland). Alternatively, cell proliferation was determined by Hoechst 33342 staining. Cells were seeded at 2 × 10^4^ cells/well in 24-well plates and treated with 0.5, 1, 2, 5 or 10 μM nintedanib in the growth medium for 1, 2, or 3 days. The drug-treated cells were fixed with 4% paraformaldehyde for 10 min after washing three times with phosphate-buffered saline (PBS, Welgene). Then, the cells were treated with 10 μg/ml Hoechst 33342 diluted in PBS for 1 hr. After removal of the Hoechst 33342 staining buffer and three washed with PBS, the fluorescence intensity of the cells at 461 nm was measured using an Infinite M200PRO (TECAN).

### Apoptosis assay

Senescent and nonsenescent HDFs were seeded at 35 × 10^5^ cells in 100-mm dishes. After an overnight incubation, the cells were treated with nintedanib (5 μM) or ABT263 (5 μM) for 3 days. The cells were harvested and washed three times with PBS. Then, the harvested cells were resuspended in 100 μl of 1× Annexin V binding buffer (0.1 M HEPES (pH 7.4), 1.4 M NaCl, and 25 mM CaCl_2_) containing 5 μl of Annexin V-Alexa Fluor®350 conjugate (Thermo Fisher Scientific, A23202) and 5 μl of 100 μg/ml propidium iodide (PI). After 30 min of incubation in the dark, 400 μl of 1× Annexin V binding buffer was added (final volume: 500 μl) to each sample. The cells were analyzed using a flow cytometer (LSR Fortessa, BD) within 1 hr to determine the intensity of Annexin V staining (excitation wavelength of 488 nm with a 530/30 nm bandpass filter, DAPI channel) and that of PI (excitation wavelength of 561 nm with a 583/22 nm bandpass filter, PE channel). The results were analyzed using FlowJo (version 10).

### Cell cycle assay

Nonsenescent HDFs were seeded at 35 × 10^5^ cells in 100-mm dishes a day prior to nintedanib (5 μM) treatment. After one day of drug treatment, the cells were harvested and washed three times with PBS. Then, the cells were fixed in 70% ethanol overnight at −20 °C and stained with a PI staining solution (5 μg/ml PI and 0.5 μg/μl RNase A in PBS). The DNA content was determined by flow cytometry (LSR Fortessa, BD) (excitation wavelength of 561 nm with a 583/22 nm bandpass filter, PE channel). The results were analyzed using FlowJo (version 10).

### Transfection of siRNA into senescent HDFs

Senescent HDFs were seeded at 5 × 10^4^ cells/well in 6-well plates and incubated overnight. Scramble siRNA or siRNA-STAT3 was transfected into cells using Lipofectamine RNAiMAX reagent (Thermo Fisher) according to the manufacturer’s instructions. After 3 days of incubation, the cells were harvested. Lysates were prepared from the siRNA-transfected cells with 1× Laemmli sample buffer (#161-0737, Bio–Rad Laboratories, Hercules, CA) containing 5% β-mercaptoethanol. STAT3 expression knockdown was evaluated by western blot analysis using an anti-STAT3 antibody (#9145, Cell Signaling Technology). The siRNA sequences were as follows: siRNA-STAT3: antisense: CAG CAA AAA GUU UCC UAC A, sense: UGU AGG AAA CUU UUU GCU G; and scramble-siRNA: antisense: UCGAACUAUGCUGUUUCGATT, sense: AGC UUG AUA CGA CAA AGC UTT.

### Western blot analysis

Cell lysates were prepared by resuspending cell pellets in 1× Laemmli sample buffer containing 5% β-mercaptoethanol. Protein from lung tissues were extracted using IPH lysis buffer (50 mM Tris (pH 8.0), 150 mM NaCl, 5 mM EDTA, 0.5% NP40) containing protease inhibitor cocktail (Roche). The protein lysates were separated by SDS–PAGE and then electrotransferred to nitrocellulose membranes (Millipore Corp., Bradford, MA, USA). Detection of specific proteins was carried out with enhanced chemiluminescence reagents following the manufacturer’s instructions (P90720, Millipore Corporation, MA, USA). The primary antibodies used for western blotting were as follows: anti-Caspase-9 (#9508), anti-Caspase-8 (#9746), anti-Caspase-7 (#8438), anti-Caspase-3 (#9664), anti-Bim (#2933), anti-Bak (#3814), anti-Bcl-xL (#2762), anti-p-Jak2 (#3771), anti-Jak2 (#3230), and anti-p-STAT3^Y705^ (#9145), which were purchased from Cell Signaling Technology. Anti-CDK2 (sc-6248), anti-p53 (sc-126), anti-STAT3 (sc-8019), anti-PARP-1 (sc-74470), anti-Cyclin D1 (sc-8396), and anti-β-actin (sc-47778) antibodies were purchased from Santa Cruz Biotechnology. An anti-p21 (ab7960), anti-p16 (ab108349), anti-IL-6 (ab6672), and anti-TNF-α (ab6671) antibodies were purchased from Abcam. Densitometry analyses were performed using ImageJ software (National Institutes of Health, NIH).

### Animals

Male C57BL/6 J mice were obtained from Hyochang Science (Daegu, Republic of Korea). Animal studies were approved by the Institutional Animal Care and Use Committee of the College of Medicine, Yeungnam University (YUMC-AEC2020-010). Animals were housed in a light- and temperature-controlled room and had free access to drinking water and a standard rodent diet. To induce in vivo IPF model, male C57BL/5 J mice (8 weeks old) were anesthetized and underwent intratracheal instillation of PBS or 2 U/kg bleomycin [25]. After 7 days, nintedanib (0.5396 mg/kg) or vehicle was administered intraperitoneally three times per week for 24 days (vehicle: *n* = 3; bleomycin: *n* = 2; bleomycin+ABT263: *n* = 3; and bleomycin + nintedanib: *n* = 4). The mice were sacrificed 28 days after intratracheal instillation. Lung tissues were harvested and analyzed. All mice used in the experiment were included in the analysis. All randomization was carried by investigators. No blinding was done for drug administration.

### Histological, immunohistochemical and immunofluorescence assessment

Lung tissues were collected on 24 days after bleomycin instillation. Tissues were fixed overnight with 10% (w/v) formaldehyde solution (HT501128, Sigma-Aldrich), dehydrated using graded alcohol, and embedded in paraffin wax. Four micro meter lung tissue sections were stained with Hematoxylin and eosin (H&E) to assess the lung interstitial damage, with Masson’s trichrome (collagen stains blue) to detect collagen deposition [26, 27]. To immunofluorescence analysis, ten micro meter lung tissue section were stained with antibody. The following antibodies have been used for immunofluorescence staining: anti-p53 (sc-126, Santa Cruz Biotechnology), anti-p16 (ab81278, Abcam)

### Senescence-associated β-galactosidase (SAβG) staining

A SAβG activity in cells was assessed by staining with senescence-β-galactosidase staining kit (#9860, Cell Signaling Technology) as previously described [28]. Cells were imaged after 24 h using a light microscope (OLYMPUS CKX41) at 10x magnification. Lung tissues were collected on 24 days after bleomycin instillation. Tissues were fixed overnight with 10% (w/v) formaldehyde solution (HT501128, Sigma-Aldrich), dehydrated using graded alcohol, and embedded in paraffin wax. Four micro meter lung tissue were stained with senescence-β-galactosidase staining kit (#9860, Cell Signaling Technology) to measure senescence levels. The immune fluorescence SAβG activity in frozen tissue was measured by staining with X-gal as previously described or with SPiDER-βGal (Dojindo Molecular Technologies, Inc., Kumamoto, Japan) as previously described [29].

### Plasmids, cloning, mutagenesis and lentiviral particles

Expression plasmids for Myc-tagged human STAT3 was purchased from Origene (RC215836). The PCR-based site-directed mutagenesis for generating STAT3-Y705E (constitutively active STAT3) [30] and STAT3-Y705F (dominant negative mutant) [31] were performed with the following primers. STAT3-Y705E-F: 5ʹ-ACCCAGGTAGCGCTGCCCCAgagCTGAAGACCA-AGTTTATCTG-3ʹ and STAT3-Y705E-R: 5ʹ-CAGATAAACTTGGTCTTCAGctcTGGGGC-AGCGCTACCTGGGT-3. STAT3-Y705F-F: 5ʹ-CCAGGTAGCGCTGCCCCATtCCTGAA-GACCAAGTTTATC-3ʹ and STAT3-Y705F-R: 5ʹ-GATAAACTTGGTCTTCAGGaATGGG-GCAGCGCTACCTGG-3ʹ. Then individual STAT3 genes were cloned into between AsiSI and XhoI of pLenti6.3 vector to produce lentivial particles. Lentiviruses were produced in HEK-293T cell (ATCC, CRL-3216) by co-transfecting the lentiviral expression vector, pCMV-VSV-G envelope plasmid (Addgene, Cambridge, MA), and psPAX2 packaging plasmid (Addgene, Cambridge, MA) with Lipofectamine 2000 (Invitrogen) according to the manufacturer’s instructions. Lentiviral particles containing LacZ gene were used as control.

### Transcriptome analysis

Transcriptome analysis was performed by Macrogen Inc. In detail, the total RNA concentration was calculated by Quant-IT RiboGreen (Invitrogen, #R11490). To assess the integrity of total RNA, samples were run on a TapeStation RNA ScreenTape (Agilent, #5067-5576). Only high-quality RNA preparations with an RNA integrity number (RIN) greater than 7.0 were used for RNA library construction. Libraries were independently prepared with 1 µg of total RNA for each sample with an Illumina TruSeq Stranded mRNA Sample Prep Kit (Illumina, Inc., San Diego, CA, USA, #RS-122-2101). The first step in the workflow involved purifying the poly‐A-containing mRNA molecules using poly‐T‐attached magnetic beads. Following purification, the mRNA molecules were fragmented into small pieces using divalent cations under elevated temperature. The cleaved RNA fragments were reverse transcribed into first-strand cDNA using SuperScript II reverse transcriptase (Invitrogen, #18064014) and random primers. This was followed by second-strand cDNA synthesis using DNA Polymerase I, RNase H and dUTP. These cDNA fragments then underwent an end repair process, the addition of a single ‘A’ base, and adaptor ligation. The products were then purified and amplified with PCR to create the final cDNA library. The libraries were quantified using KAPA Library Quantification kits for Illumina Sequencing platforms according to the qPCR Quantification Protocol Guide (KAPA BIOSYSTEMS, #KK4854) and qualified using a TapeStation D1000 ScreenTape (Agilent Technologies, #5067-5582). Indexed libraries were then run on an Illumina NovaSeq (Illumina, Inc., San Diego, CA, USA), and paired-end (2 × 100 bp) sequencing was performed. The raw reads from the sequencer were preprocessed to remove low quality and adapter sequence before analysis and aligned the processed reads to the *Homo sapiens(hg38)* using HISAT v2.1.0 [32]. The reference genome sequence of *Homo sapiens(hg38)* and annotation data were downloaded from the UCSC table browser (http://genome.uscs.edu). After alignment, StringTie v2.1.3b [33, 34] was used to assemble aligned reads into transcripts and to estimate their abundance.

### Statistical analysis of gene expression level

Genes with one more than zeroed Read Count values in the samples were excluded. To facilitate log2 transformation, 1 was added to each Read Count value of filtered genes. Filtered data were log2-transformed and subjected to RLE normalization. Statistical significance of the differential expression data was determined using nbinomWaldTest using DESeq2 and fold change in which the null hypothesis was that no difference exists among groups. False discovery rate (FDR) was controlled by adjusting p value using Benjamini-Hochberg algorithm. For DEG set, hierarchical clustering analysis was performed using complete linkage and Euclidean distance as a measure of similarity. Gene-enrichment and functional annotation analysis and pathway analysis for significant gene list were performed based on DAVID v6.8, https://david.ncifcrf.gov) [35]. The Gene Set Enrichment Analyses (GSEA) algorithm determines whether the genes in a specific gene set are over-represented (enrichment) at the top or at the bottom of a background gene list which is sorted based on specified statistics of interest. GSEA calculates the enrichment score (ES) [36] which is then normalized to mean enrichment of random samples of the same gene set size. The statistical significance of enrichment was determined using permutation-based analysis (*n* = 1000). A positive/negative normalized ES (NES) will indicate that genes in specific gene sets tend to be more located at the top/bottom of a reference ranked gene list. GSEA was performed using the Bioconductor R package clusterProfiler (v3.14.3) [37].

### Statistical analyses

All data were analyzed using ANOVA with GraphPad Prism 7 software. Post-hoc analyses were completed with Tukey’s multiple comparison test with *p* < 0.05 set as the significance level. Data are shown as the mean ± S.D. (*p < 0.05, **p < 0.01, and ***p < 0.001).

## Results

### Nintedanib induces selective cytotoxicity in senescent human dermal fibroblasts (HDFs)

**Fig. 1:**
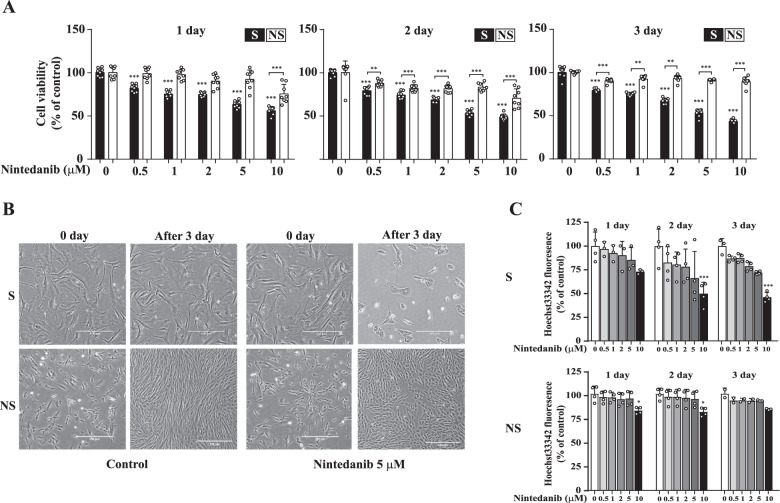
Nintedanib reduces the viability of senescent HDFs. **A** Senescent (S) HDFs and nonsenescent (NS) HDFs were treated with nintedanib (0.5, 1, 2, 5, and 10 μM) for 1, 2, or 3 days, and then CCK-8 assays were performed to assess cell viability. *n* = 8. **B** Cell morphological changes after three days of treatment with DMSO (control) or nintedanib (5 μM). Senescent (S) HDFs (5 × 10^4^ cells per well) and nonsenescent (NS) HDFs (8 × 10^4^ cells per well) were separately plated in 6-well plates. Images were randomly captured by inverted microscopy. Scale bar, 500 μm. **C** Senescent (S) HDFs and nonsenescent (NS) HDFs were treated with DMSO or nintedanib (0.5, 1, 2, 5, and 10 μM) for three days, and Hoechst 33342 staining was then performed to investigate cell growth. The data normalized to those for DMSO-treated cells are shown as the mean ± S.D. *p < 0.05, **p < 0.01, ***p < 0.001 by one-way ANOVA with Tukey’s post hoc test.

Our previous studies confirmed the effectiveness of senotherapeutic candidates derived from a pool of kinase inhibitors of senescent fibroblasts in a replicative senescence (RS) model [24, 38]. Considering these results, we selected the initial candidates on the basis of their senolytic activity manifested by their abilities to induce cytotoxicity and increase reactive oxygen species (ROS) levels. Then, we reduced the candidate list by reviewing publications on the cell physiology and side effects related to each compound in preclinical studies [16]. Finally, we selected nintedanib, an FDA-approved receptor tyrosine kinase inhibitor, as a potential senolytic and, performing a cell counting kit-8 (CCK-8) assay, evaluated its ability to induce selective cell death based on the senescent state of HDFs. In agreement with the screening results, nintedanib exhibited greater cytotoxicity in senescent HDFs than in nonsenescent HDFs at the tested concentration (Fig. 1A). Moreover, the difference in cytotoxicity between senescent HDFs and nonsenescent cells was increased upon extended exposure to the drug. In particular, after three days of exposure to nintedanib at a concentration of 5 or 10 μM, the difference in cell viability was more than 40% (Fig. 1A). Subsequent experiments to determine senolytic mode of action of nintedanib were performed after a 3-day 5 μM nintedanib treatment of HDFs. In agreement with the CCK-8 assay results showing nintedanib-induced selective cytotoxicity in senescent cells, nintedanib almost completely eliminated the senescent HDFs (Fig. 1B, top panel). Interestingly, nonsenescent HDFs treated with nintedanib showed retarded cell proliferation (Fig. 1B, bottom panel).

Cell viability measured by CCK-8 assay is based on formazan dye formation by cellular dehydrogenases, which primarily reflects cellular metabolic activity [39]. Because metabolic activity can be influenced by the inhibition of kinases and since the metabolism of senescent cells differs substantially from that of proliferating cells, we evaluated the dose-dependent inhibition of cell proliferation caused by nintedanib using chromosomal DNA-based Hoechst 33342 staining. Consistent with the results of the CCK-8 assay and morphological changes seen after nintedanib treatment, nintedanib (5 or 10 μM) reduced senescent HDF proliferation or survival by approximately 30–50% (Fig. 1C, top panel), and the proliferation of nonsenescent HDFs was slightly decreased (approximately 5–15% at 10 μM) without a significant time-dependent reduction in the cell number after 3 days of nintedanib exposure (Fig. 1C, bottom panel). These results indicated that nintedanib was effective as a senolytic and affected cell survival through different modes depending upon the senescent state of HDFs.

### Nintedanib induces apoptotic cell death in senescent cells

**Fig. 2:**
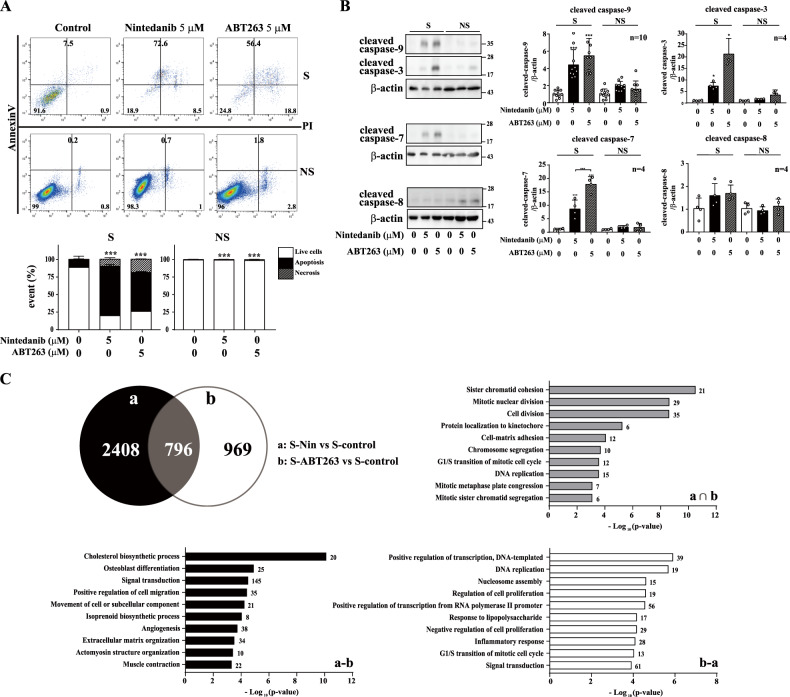
Nintedanib induces apoptotic cell death in senescent HDFs. **A** Senescent (S) and nonsenescent (NS) HDFs were treated with DMSO, nintedanib (5 μM), or ABT263 (5 μM) for three days, and flow cytometry analysis was performed using Annexin V/PI staining to identify apoptotic cells. *n* = 4. **B** Senescent (S) and nonsenescent (NS) HDFs were treated with DMSO, nintedanib (5 μM), or ABT263 (5 μM) for three days, and then western blot assays using anti-caspase-9, anti-caspase-3, anti-caspase-7, and anti-caspase-8 antibodies were performed to investigate the apoptotic pathways involved in the senolytic effect. *n* = 4–10. **C** Venn diagram (top left) showing the number of differentially expressed genes (DEGs) in senescent cells treated with nintedanib (Group a) or treated with ABT263 (Group b). A Gene Ontology biological process (GOBP) analysis (top right, bottom left and bottom right) of each set (a∩b, a-b, and b-a, respectively). The data normalized to those for DMSO-treated cells are shown as the mean ± S.D. *p < 0.05, **p < 0.01, ***p < 0.001 by one-way ANOVA with Tukey’s post hoc test.

Most senolytic compounds that have been identified to date selectively induce apoptosis in senescent cells. To investigate whether the decreased cell viability induced by nintedanib is due to apoptotic cell death, we treated HDFs with nintedanib (5 μM) or ABT263 (5 μM) for three days and then carried out an apoptosis assay by costaining with Annexin V and propidium iodide (PI). As expected, nintedanib induced apoptotic cell death in majority (72.6%) of senescent HDFs, compared with the effect on non-treated senescent HDFs (7.5%) (Fig. 2A). The selective induction of apoptosis in senescent cells by nintedanib was similar to that induced by ABT263 (Fig. 2A). Interestingly, the expression of Bcl-xL was inhibited by ABT263 but, not nintedanib (Supplementary Fig. 1A). Moreover, Bim, a proapoptotic factor, was induced by only nintedanib, not by ABT263, while Bak and cleaved PARP-1 were not affected by either drug (Supplementary Fig. 1B, C). These results strongly suggest that nintedanib induces apoptosis in a somewhat different manner than that induced by ABT263.

Apoptosis is induced by two distinct pathways: the caspase-8-mediated extrinsic pathway and the caspase-9-mediated intrinsic pathway [40]. Nintedanib activated the cleavage of caspase-9 but not that of caspase-8, which indicated that nintedanib induced apoptotic cell death through the intrinsic pathway (Fig. 2B). The cleavage of caspase-9 and the downstream factors caspase-3 and caspase-7 after nintedanib treatment was prominent in senescent cells compared with nonsenescent cells (Fig. 2B). Interestingly, ABT263 induced more cleavage of caspases in nonsenescent HDFs than did nintedanib (Fig. 2B). These data collectively indicate that nintedanib causes senolytic effects through caspase-9-mediated intrinsic apoptosis but that this compound activates caspase-9 through a mechanism different from that of ABT263, as nintedanib did not exhibit a pattern of apoptotic factor modulation identical to that exhibited by ABT263.

To further examine the different senolytic modes of action between nintedanib and ABT263, we carried out a transcriptome analysis of senescent cells treated with nintedanib or ABT263. We identified 3204 (Group a) and 1765 (Group b) differentially expressed genes (DEGs), including 796 common DEGs, in nintedanib- and ABT263-treated senescent HDFs, respectively, compared with DMSO-treated senescent cells (Fig. 2C, Venn diagram). For systematic analysis of the cellular processes affected by each senolytic drug, we performed Gene Ontology biological process (GOBP) enrichment analysis of the up- and downregulated genes in senolytic agent-treated senescent cells. The DEGs commonly impacted by both senolytic drugs (Fig. 2C, a ∩ b, 796 genes) were mainly associated with cell division. Interestingly, each senolytic agent affected distinct cellular processes: the unique nintedanib-induced DEGs (Fig. 2C, a-b, 2408 genes) were associated with signal transduction, extracellular matrix organization and cell migration, while the unique ABT263-induced DEGs (Fig. 2C, b-a, 969 genes) were associated with cell proliferation and replication, positive regulation of transcription and signaling transduction. The gene enrichment analysis results were in agreement with the changes in apoptosis-regulating proteins, indicating that even though both nintedanib and ABT263 affected the expression of genes regulating cell maintenance in senescent cells, nintedanib has a mode of action for inducing senolytic effects distinct from that of ABT263.

### Nintedanib arrests the cell cycle in the G1 phase without inducing cytotoxicity in nonsenescent HDFs

**Fig. 3:**
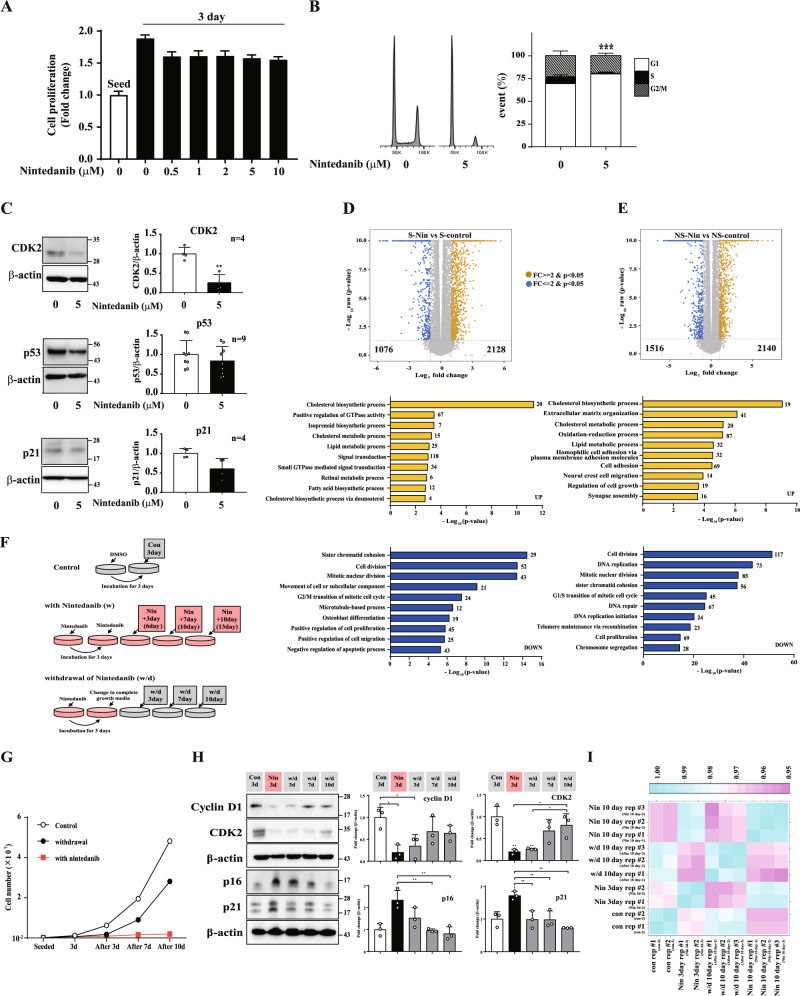
Nintedanib induces G1-phase cell cycle arrest without inducing cell death in nonsenescent HDFs. **A** Nonsenescent (NS) HDFs were treated with nintedanib (0.5, 1, 2, 5, and 10 μM) for 3 days, and then CCK-8 assays were performed to evaluate cell viability and proliferation. *n* = 8. **B** Nonsenescent (NS) HDFs were treated with DMSO or nintedanib (5 μM) for one day and flow cytometry analysis of PI staining was performed to determine the number of cells in each cell cycle phase. *n* = 5. **C** Nonsenescent (NS) HDFs were treated with DMSO or nintedanib (5 μM) for one day, and then western blot assays using anti-CDK2, anti-p53, and anti-p21 antibodies were performed to investigate the protein expression of cell cycle regulators. *n* = 4–9. **D** Volcano plot (top) and GOBP analysis (bottom) of up- (yellow) and downregulated (blue) DEGs in nintedanib-treated senescent cells. **E** Volcano plot (top) and GOBP analysis (bottom) of up- (yellow) and downregulated (blue) DEGs in nintedanib-treated nonsenescent cells. **F** Experimental scheme for testing the restoration of cell proliferation after nintedanib removal. The ‘control’ group was treated with DMSO and the ‘Nin *n* day’ group was treated with nintedanib for *n* days. The ‘withdrawal’ group was treated with nintedanib for 3 days and then cultured in complete growth medium from 3 to 10 days after drug removal. **G** Nonsenescent HDFs were treated according to the experimental scheme, and the number of cells was monitored to investigate the effect of drug removal on cell proliferation. **H** Nonsenescent HDFs were treated according to the experimental scheme, and then western blot assays using anti-cyclin D1, anti-CDK2, anti-p16, and anti-p21 antibodies were performed to investigate the restoration of cell proliferation after drug removal. *n* = 3. **I** Correlation matrix for the transcriptome of all samples shown in **G**. The data normalized to those for DMSO-treated cells are shown as the mean ± S.D. *p < 0.05, **p < 0.01, ***p < 0.001 by one-way ANOVA with Tukey’s post hoc test.

We noticed that nintedanib affected cell cycle arrest rather than apoptotic cell death in nonsenescent HDFs (Fig. 1B, C). Although nintedanib reduced the capacity to increase the cell population 3 days post-treatment, the absolute cell number was higher than the initial number of cells seeded, which supported the supposition that nintedanib delays the cell cycle but does not induce death in nonsenescent cells (Fig. 3A). In agreement with the cell proliferation assay results, the proportion of nonsenescent cells in the G1 phase of the cell cycle was increased one day post-treatment, and the expression of CDK2, which regulates the G1-to-S phase transition, was significantly reduced (Fig. 3B, C). Interestingly, the levels of p53 and p21, which are CDK inhibitors, were not increased by nintedanib (Fig. 3C).

To investigate the different actions of nintedanib according to the cellular senescence status, we performed a global transcriptome analysis of senescent and nonsenescent cells treated with nintedanib. We identified 2128 upregulated genes and 1076 downregulated genes in senescent HDFs treated with nintedanib compared with DMSO-treated senescent HDFs (Fig. 3D, volcano plot). In nonsenescent HDFs treated with nintedanib, 2140 upregulated genes and 1516 downregulated genes were identified (Fig. 3E, volcano plot). For systematic analysis of biological processes affected by the senescence-dependent effects of nintedanib, we performed a GOBP enrichment analysis of the up- and downregulated genes in the cells in each senescent state after drug treatment. It was not surprising that the downregulated genes were mainly associated with cell proliferation and cell division, regardless of cellular senescence status (Fig. 3D, E, DOWN), which represents the nintedanib-induced cell cycle arrest in the G1 phase observed in nonsenescent cells. Interestingly, the upregulated genes in nintedanib-treated senescent HDFs were mainly associated with metabolic processes and signal transduction, while the genes in nonsenescent cells were associated with extracellular matrix organization, cell adhesion and regulation of cell growth, as well as metabolic processes (Fig. 3D, E, UP).

Next, to determine whether the cell cycle of nonsenescent cells is temporarily stopped by nintedanib, we tested the restoration of cell proliferation after the withdrawal of nintedanib from nonsenescent HDFs. As expected, the cell population exponentially increased 3 days after the withdrawal of nintedanib from nonsenescent HDFs (Fig. 3G, withdrawal), an outcome similar that of the control. However, the cells continuously treated with nintedanib maintained cell proliferation arrest but did not induce cell death (Fig. 3G, with nintedanib). In agreement with the reactivation of cell proliferation after drug removal, the expression of cyclin D1 and CDK2 was increased 7 days post drug withdrawal compared with the level of these proteins in the presence of nintedanib (Fig. 3H). Correspondingly, the levels of p16 and p21 gradually decreased 3 days post drug removal (Fig. 3H). The altered cell morphology by nintedanib was also recovered 7 days post drug removal (Supplementary Fig. 2). A transcriptome analysis confirmed that the overall transcript expression in nonsenescent cells after the withdrawal of nintedanib was much more similar to that in proliferating nonsenescent cells (control), and was district from nintedanib-treated nonsenescent cells (Fig. 3I). Collectively, these data indicated that nintedanib arrests the cell cycle without inducing cytotoxicity in nonsenescent as a reversible manner. These results suggest that nintedanib might have two different actions: to induce cell death in senescent cells and to induce cell cycle arrest but to keep their viability via regulating cell adhesion and growth in nonsenescent cells.

### Nintedanib induces cell death in senescent cells by suppressing JAK2/STAT3 pathway

As mentioned above, nintedanib functions as a senolytic. To investigate the mode of action of this drug, we performed a Kyoto Encyclopedia of Genes and Genomes (KEGG) pathway analysis based on the transcriptome of nintedanib-treated senescent cells (senescent HDFs-Nin) and DMSO-treated senescent cells (senescent HDFs-con). The results showed that various signaling pathways, including the JAK-STAT, MAPK, and Wnt signaling pathways, were enriched (Supplementary Fig. 3A). Among them, JAK-STAT3 signaling pathway is known crucial pathway in cell growth and death, cell division, and proliferation [41–43]. In particular, previous studies have shown that nintedanib modulates STAT3 activity, resulting in the apoptosis of triple-negative breast cancer cells [19] as well as ameliorates IPF by inhibiting the JAK2/STAT3 pathway [44]. Of note, it has been known that STAT3 regulates bleomycin-induced lung fibrosis [45]. Furthermore, a gene set enrichment analysis (GSEA) with JAK2 pathway-related genes showed that the JAK2/STAT3 pathway appears to be more correlated with transcriptional alternation in nintedanib-treated senescent HDFs than with ABT263-treated senescent cells (Supplementary Fig. 3B, C). Therefore, we focused on the JAK-STAT3 pathway among the various KEGG-enriched signaling pathways, although we could not exclude another signaling involving nintedanib-induced apoptosis in senescent cells.

**Fig. 4:**
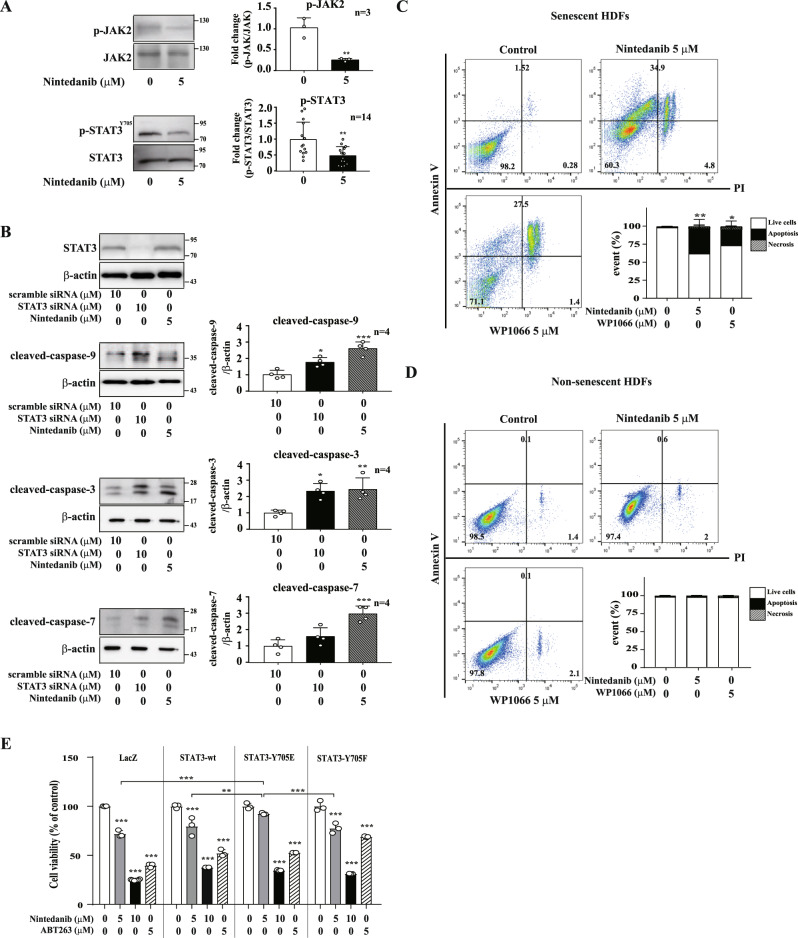
Nintedanib inhibits tyrosine phosphorylation of JAK2 and STAT3 in senescent HDFs. **A** Senescent (S) HDFs were treated with DMSO or nintedanib (5 μM) for one hour, and then western blot assays using anti-p-JAK2, anti-JAK2, anti-p-STAT3, and anti-STAT3 antibodies were performed to identify the signaling pathways affected by nintedanib. **B** Senescent (S) HDFs were transfected with scramble siRNA or STAT3-specific siRNA three days prior to nintedanib (5 μM) treatment. Then, after drug exposure for three days, western blot assays using anti-STAT3, anti-caspase-9, anti-caspase-3, and anti-caspase-7 antibodies were performed to evaluate apoptosis induction. **C** Senescent (S) HDFs were treated with DMSO, nintedanib (5 μM), or WP1066 (5 μM, a STAT3 kinase inhibitor) for three days, and a flow cytometry analysis with Annexin V/PI staining was performed to identify apoptotic cells. **D** Nonsenescent (NS) HDFs were treated with DMSO, nintedanib (5 μM), or WP1066 (5 μM, STAT3 kinase inhibitor) for three days, and flow cytometry analysis with Annexin V/PI staining was performed to identify apoptotic cells. **E** Nonsenescent HDFs were infected with lentiviral particles having LacZ (as control), STAT3-wt, STAT3-Y705E, or STAT3-Y705F encoded gene and then CCK-8 assays were performed after nintedanib treatment for 3 days to assess cell viability. *n* = 3. The data normalized to those for DMSO-treated cells are shown as the mean ± S.D. *p < 0.05, **p < 0.01, ***p < 0.001 by one-way ANOVA with Tukey’s post hoc test.

To investigate whether the senolytic effect of nintedanib is mediated via STAT3 inhibition, we examined the effect of nintedanib on the phosphorylation of JAK2 and STAT3 in senescent HDFs. Consistent with the previously published results for breast cancer cells, nintedanib reduced the phosphorylation of JAK2 and STAT3 in senescent HDFs (Fig. 4A). To ensure that the regulation of STAT3 is associated with triggering apoptosis in senescent cells, we investigated the STAT3-dependent cell viability of senescent cells. Knocking down STAT3 expression with siRNA induced the activation of cleaved-caspase-9, caspase-3, and caspase-7, which are involved in the intrinsic pathway of apoptosis, similar to the effect of nintedanib (Fig. 4B). In addition, WP1066 (5 μM), a STAT3 inhibitor, increased the population of Annexin-V/PI-costained senescent HDFs (Fig. 4C), but exerted no effect on nonsenescent HDFs (Fig. 4D).

To confirm that the senolytic effect of nintedanib is mediated by STAT3 inhibition, we tested the nintedanib-induced senolytic effect with wild-type STAT3, STAT3-Y705F, a dominant-negative active STAT3 mutant or STAT3-Y705E, a constitutively active mutant; all three constructs were overexpressed via lentiviral transduction. (Supplementary Fig. 4). The results of a CCK-8 cell viability assay after nintedanib exposure for 3 days showed that STAT3-Y705E-overexpressing cells exhibited increased resistance to nintedanib-induced cytotoxicity compared with that of STAT3-wt or STAT3-Y705F-overexpressing cells (Fig. 4E). These data support the hypothesis that the senolytic effect of nintedanib is mediated by STAT3 inhibition.

### Nintedanib reduces the number of SAβG-positive senescent cells in bleomycin-induced lung fibrosis

**Fig. 5:**
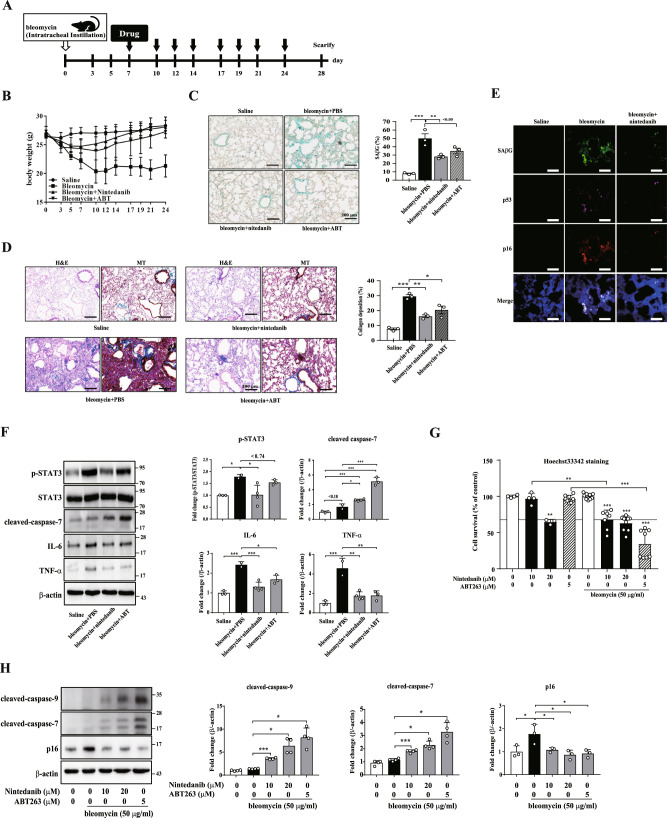
Nintedanib shows senolysis in bleomycin-induced in vivo and in vitro model. **A** Experimental scheme for establishing bleomycin-induced lung fibrosis model: saline (*n* = 3), PBS (bleomycin; *n* = 2), ABT263 (bleomycin + ABT; *n* = 3), or nintedanib (bleomycin + nintedanib; *n* = 4) was administrated via intraperitoneal injection three times per week for 24 days (black arrow). Control mice were treated with PBS are labeled saline (*n* = 3). **B** Body weight changes after bleomycin followed by drug treatment. **C**, **D** Lung tissues were stained with an SAβG staining kit to measure the number of senescent cells (**C**), hematoxylin and eosin (H&E) to assess lung interstitial damage, and Masson’s trichrome (MT) staining (collagen is stained blue) to detect collagen deposition (**D**). Scale bar, 200 μm. **E** Lung tissues were stained with SPiDER-βGal to measure SAβG activity and with anti-p16 and anti-p53 antibodies to determine whether senescence was attenuated. Scale bar, 200 μm. **F** Western blot assays using anti-p-STAT3, anti-STAT3, anti-caspase-7, anti-IL-6 and anti-TNF-α antibodies were performed to identify the apoptotic pathways involved in the lung tissue. **G** Bleomycin-induced senescent HPFs was treated with nintedanib or ABT263 for 3 days. Then, Hoechst 33342 staining was performed to assess cell viability. *n* = 3 **H** Bleomycin-induced senescent HPFs were treated with nintedanib or ABT263 for 3 days, and then, western blot assays using anti-caspase-9, anti-caspase-7, and anti-p16 antibodies were performed to identify the apoptotic pathways involved in the senolytic effect. The data normalized to those for DMSO-treated cells are shown as the mean ± S.D (**F**, **G**). *p < 0.05, **p < 0.01, ***p < *0.001* by one-way ANOVA with Tukey’s post hoc test.

The accumulation of senescent cells plays a critical role in lung fibrosis models [25, 46]. Therefore, we investigated the effects of nintedanib on senescent cells in a mouse model of bleomycin-induced lung fibrosis (Fig. 5A). Because body weight loss is a key indicator of pathological severity in bleomycin-treated lung injury [25, 47], we first measured the change in the body weight of mice treated with senolytic drugs. As expected, the administration of nintedanib ameliorated bleomycin-treated body weight loss (Fig. 5B), similar to the effect of ABT263. In particular, the animals with body weight recovery after nintedanib administration showed a significant reduction in the number of SAβG-positive cells in damaged lung tissues, similar to the level observed in tissue from ABT263-treated animals (Fig. 5C). Additionally, nintedanib administrated animals mitigated the bleomycin-induced collagen deposition similar to that observed in ABT263-treated animals (Fig. 5D). Correspondingly, at the molecular level, nintedanib treatment downregulated the protein expression of CDK inhibitors, including p53 and p16 (Fig. 5E) and inhibited the phosphorylation of STAT3 (Fig. 5F) in bleomycin-treated lung tissue. Nintedanib treated tissues also showed reduced expression of IL-6 and TNF-α, known as SASPs, and increased level of cleaved caspase-7 (Fig. 5F), which would be expected as a consequence of nintedanib-induced senolysis. To test the senolytic effect of nintedanib on cells under mimicked in vivo conditions, we analyzed bleomycin-treated human lung fibroblasts (HPFs). At the concentration of bleomycin that induced cellular senescence in HPFs (Supplementary Fig. 5A and 5B), nintedanib (10 μM) showed a senolytic effect, although it needed a higher concentration of the drug than the effective concentration (5 μM) on replicative senescent HDFs (Fig. 5G and Supplementary Fig. 5C). In agreement with these data, nintedanib induced the cleavage of caspase-9 and -7, and suppressed the expression of p16 (Fig. 5H), indicating that nintedanib induces a senolytic effect via the apoptotic cell death in HPFs as well. Taken together, these data demonstrate that nintedanib has a senolytic effect in vivo as well, which also implies that nintedanib, as a senolytic, is an effective drug for IPF treatment via modulating STAT3 pathway.

## Discussion

Cellular senescence is characterized by the cessation of cell division in response to various cellular defects, including DNA damage accumulation, telomere shortening, and dysfunctional subcellular organelles [48, 49]. The accumulation of senescent cells in tissues has negative effects on the structural integrity and plasticity in the environment in which the senescent cells exist, which is a major contributor to aging and age-related diseases, such as fibrosis and chronic inflammation. Therefore, developing senolytics that are effective in selectively eliminating senescent cells could be a potential strategy to delay the aging process and ameliorate the age-related phenotypes of diseases. Moreover, expanding the toolkits for senolytics affecting various cell death pathways could improve the management of the diverse statuses of aging with reduced side effects.

Senescent cells have the distinct feature of elevated antiapoptotic signaling, so the strategy for developing senolytics, including ABT263 and the combination of dasatinib and quercetin, has mainly focused on attenuating the activity or expression of antiapoptotic factors [9, 50, 51]. Our data showed that nintedanib initiated apoptosis via an intrinsic apoptotic pathway, which was similar to ABT263, a well-known senolytic that acts as an inhibitor of the Bcl-2 family (Fig. 2). However, nintedanib showed a mode of action for cell death induction distinct from that of ABT263, as Bcl-xL expression was not significantly changed by nintedanib. Additionally, Bim was induced by only nintedanib, not ABT263 (Supplementary Fig. 1). Moreover, in the transcriptome analysis, nintedanib and ABT263 produced distinct gene set enrichment patterns for their corresponding senolytic effect (Fig. 2C). Our findings suggest that nintedanib would be categorized into a novel class of senolytics.

Nintedanib, which was determined to be a senolytic in this study, is a medication used to treat IPF [17, 52] and non-small cell lung cancer (NSCLC) [53]. It is known that nintedanib rescues IPF via regulation of the JAK2/STAT3 pathway [44] and induces apoptosis by inhibiting STAT3 in triple-negative breast cancer cells [19]. Recent studies have also confirmed that C-188-9 (a STAT3 inhibitor) [45] and JSI-124 (a dual inhibitor of JAK2 and STAT3) [44] decrease pulmonary fibrosis. These previous results led us to hypothesize that the JAK2/STAT3 pathway mediates the senolytic effect of nintedanib. Our data confirmed that nintedanib-induced selective cell death is mediated by the JAK2/STAT3 signaling pathway (Fig. 4). Additionally, nintedanib administration ameliorated the SAβG-positive cell accumulation and elevated airway resistance in bleomycin-treated lungs (Fig. 5). Although other molecular mechanisms of the nintedanib-induced modulation of the JAK2/STAT3 signaling pathway could not be excluded, inactivation of receptor tyrosine kinases by nintedanib, a potent inhibitor, could be involved in this modulation. Receptor tyrosine kinases, including EGFR, regulate cell survival in human non-small cell lung cancer (NSCLC) via STAT3 activation [54]. In addition, the PDGFR-STAT3 signaling pathway is correlated with cell phenotypic transition [55]. Notably, nintedanib combined with gefitinib (an inhibitor of EGFR) abrogated the transforming growth factor β1 (TGFβ1)-induced phosphorylation of STAT3 in renal fibroblasts [56]. These results strongly support the involvement of the receptor tyrosine kinase-STAT3 axis in nintedanib-induced senolytic effects.

JAK2/STAT3 signaling is also known to be a key regulator in cellular senescence. The phosphorylation of STAT3 is increased in H_2_O_2_-induced senescent 3T3-L1 preadipocytes [57]. The regulation of STAT3 is critical for Bcl-2-interaction cell death suppressor (BIS)-targeted cellular senescence in glioblastoma [58] and ROS-induced senescence in lung fibroblasts [59]. The inhibition of STAT3 activity by a STAT3 antagonist (STA-21) was shown to reduce the p21 level and SAβG accumulation [59]. STAT3 activation in cellular senescence was also found in an in vivo system in which bone marrow-mesenchymal stem cells (BM-MSCs) from systemic lupus erythematosus (SLE) patients exhibited characteristics of senescence, abnormal activation of JAK2-STAT3 signaling, and upregulated phosphorylation of JAK2 and STAT3, and inhibition of JAK2-STAT3 improved BM-MSC senescence [60]. Taken together, these results suggest that STAT3 could be a potential target for eliminating senescent cells, which could induce recovery from lung fibrosis.

**Fig. 6:**
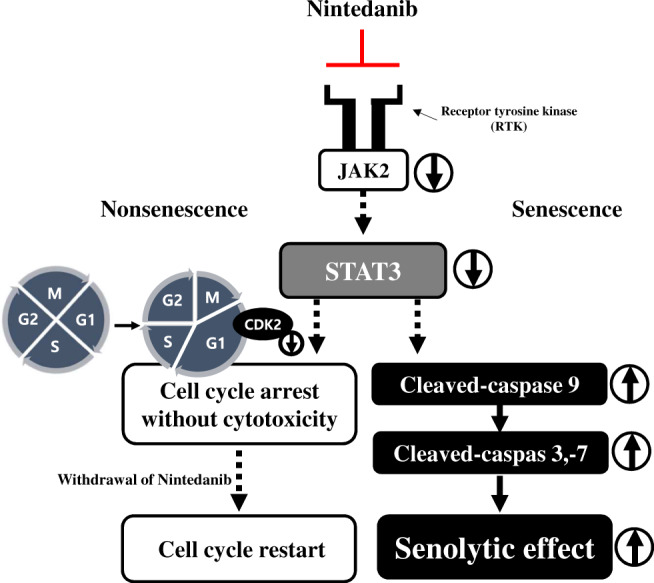
Nintedanib induces a senolytic effect in senescent HDFs via JAK2/STAT3 pathway. Proposed mechanism of senolytic effect induced by nintedanib. Nintedanib-induced senescent cell death is mediated by inhibiting STAT3 activity. Additionally, nintedanib causes cell cycle arrest in nonsenescent cells by regulating CDK2.

In this study, nintedanib induced G1 arrest of the cell cycle but did not induce cell death in nonsenescent cells. Our data showed that the cell cycle arrest induced by nintedanib might be mainly due to the downregulation of CDK2. Furthermore, the expression of CDK inhibitors, including p53 and p21, was not increased; it was even slightly decreased by nintedanib, which might maintain the proliferative capacity of nonsenescent cells even in the presence of this drug. This might be a reason that the cell population of nintedanib-treated nonsenescent HDFs still increased by 1.5-fold compared to the initially seeded population (Fig. 3). This expectation was supported by the results of the transcriptome analysis showing that nintedanib promoted the expression of genes related to cell adhesion and cell growth while inhibiting the expression of genes related to cell proliferation and the G1/S transition of the mitotic cell cycle in nonsenescent HDFs (Fig. 3E). These changes in the gene expression profile might cause cell cycle delays without inducing apoptosis in nonsenescent cells. The inhibition of cell proliferation without cytotoxic effects observed in nonsenescent cells would imply that the temporal usage of nintedanib to kill senescent cells in tissues might have a limited impact on overall tissue integrity and homeostasis. Notably, palbociclib, which induces cell cycle arrest via CDK4/6 inhibition without apoptotic cell death, is beneficial for risk management by establishing reversible bone marrow suppression in the clinic [61]. These results, together with those showing nintedanib-induced STAT3 phosphorylation inhibition, led us to propose a working model for nintedanib as a senolytic in which cell survival is controlled through different modes depending upon the senescent state of HDFs (Fig. 6).

In conclusion, we demonstrate that nintedanib is a novel senolytic agent that suppresses senescent cell survival through the JAK2/STAT3 signaling pathway. Our data also indicate that STAT3 inhibition could be a potential strategy for inducing selective cell death in senescent cells. The addition of another senolytic to the toolkit provides insights for overcoming aging or age-related diseases.

## Supplementary information

Supplementary Information

Original Western Blots

Checklist

## Data Availability

The RNA-seq dataset generated for this study was deposited in the Gene Expression Omnibus (GEO) database under accession ID GSE210020.
