# Supplementary material for: Nintedanib induces senolytic effect via STAT3 inhibition
Source: Cell Death Dis. 2022 Sep 2;13(9):760. doi: 10.1038/s41419-022-05207-8 (PMC9440251; doi:10.1038/s41419-022-05207-8)
Supplement: Supplementary file 1 — Supplementary Information [file 41419_2022_5207_MOESM1_ESM.pdf]

Supplementary Information

Nintedanib induces senolytic effect via STAT3 inhibition

Hyun-Ji Cho[1,2,*], Jeong-A Hwang[1], Eun Jae Yang[1], Eok-Cheon Kim[3], Jae-Ryong Kim[3], Sung Young Kim[4], Young Zoon Kim[5], Sang Chul Park[6], Young-Sam Lee[1,2,7,*]

[1]Department of New Biology, DGIST, Daegu 42988, Korea
[2]Well Aging Research Center, DGIST, Daegu 42988, Korea
[3]Department of Biochemistry and Molecular Biology, Smart-Aging Convergence Research Center, college of Medicine, Yeungnam University, Daegu 42415, Korea
[4]Department of Biochemistry, Konkuk University School of Medicine, Seoul 05029, Korea
[5]Division of Neuro-Oncology and Department of Neurosurgery, Samsung Changwon Hospital, Sungkyunkwan University School of Medicine, Changwon 51353, Korea
[6]The Future life and Society Research Center, Chonnam National University, Gwangju 58128, Korea
[7]New Biology Research Center, DGIST, Daegu 42988, Korea

* To whom correspondence should be addressed:

Hyun-Ji Cho, Ph.D.

Department of New Biology, DGIST, Daegu 42988, Korea
E-mail: hjcho.dr@dgist.ac.kr, Tel: +82-53-785-3307

Young-Sam Lee, Ph.D.

Department of New Biology, DGIST, Daegu 42988, Korea
E-mail: lee.youngsam@dgist.ac.kr, Tel: +82-53-785-1880

Running Title: Senolytic effect of nintedanib

This file includes:

Supplementary Figures 1-5

Supplementary Figures

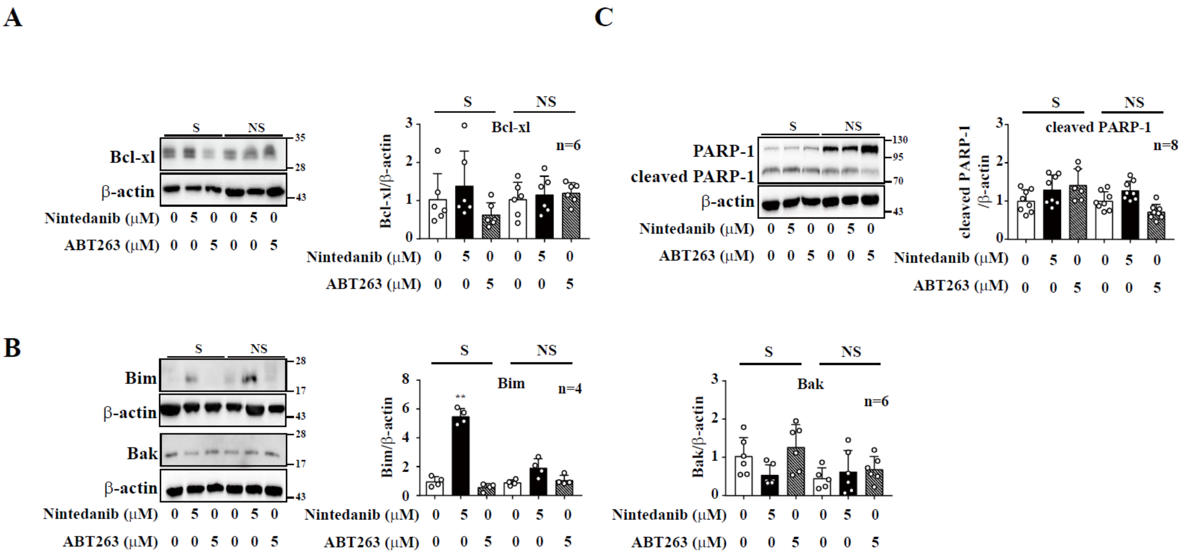

Supplementary Fig. 1. Nintedanib and ABT263 modulate apoptotic factors in HDFs via different mechanisms. A to C Senescent (S) and nonsenescent (NS) HDFs were treated with DMSO, nintedanib (5 μM), or ABT263 (5 μM) for three days, and then western blot assays using anti-Bcl-xL (**A**), anti-Bim, anti-Bak (**B**), and anti-PARP-1 (**C**) antibodies were performed to determine the expression of apoptotic factors. The data normalized to those for DMSO-treated cells are shown as the mean ± S.D. *$p<0.05$, **$p<0.01$, ***$p<0.001$ by one-way ANOVA with Tukey's post hoc test.

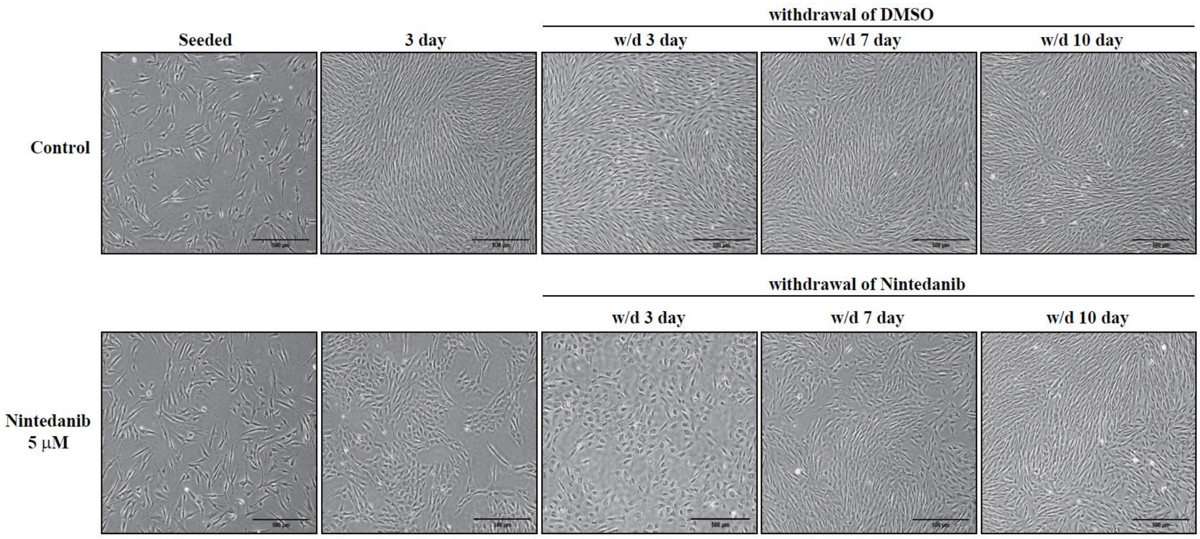

Supplementary Fig. 2 Nintedanib-induced arrested cell proliferation in nonsenescent HDFs is restored after the drug withdrawal. Cell morphological changes after nintedanib removal. DMSO or nintedanib (5 μM) was treated for the first 3 days, and then, the HDFs were cultured in complete growth medium from 3 to 10 days after chemical removal. Scale bar, 500 μm.

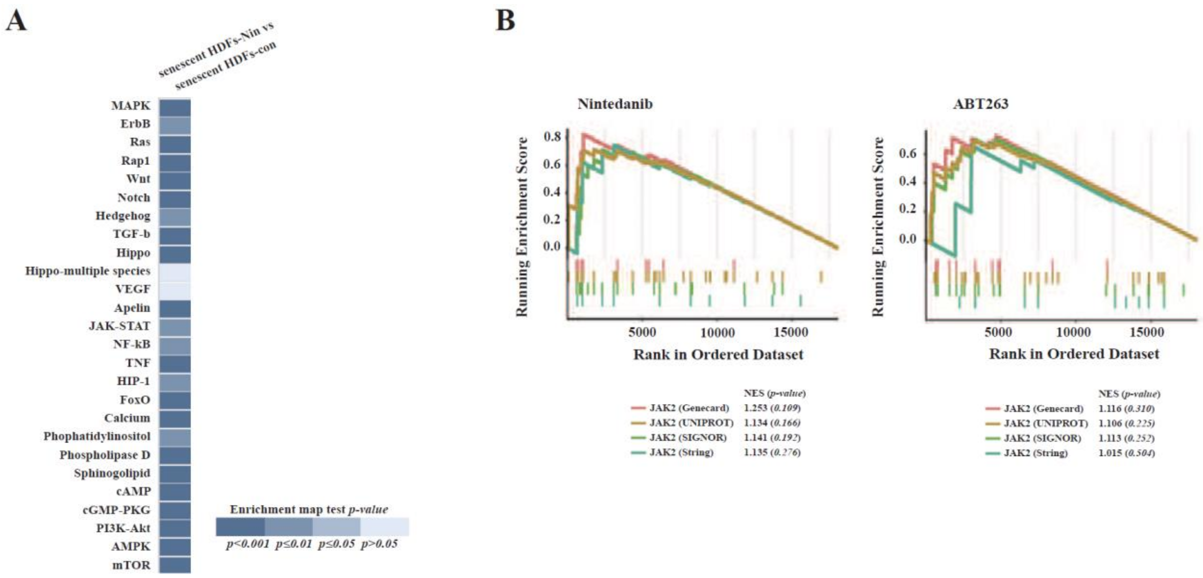

C

Gene list					
**Genecard**	**UNIPROT**		**SIGNOR**		**String**
EPOR	SLC40A1	LYN	MAPK3	CSF2RB	STAT5A
LYN	IL5RA	SIRPA	IL6ST	STAT5A	LEPR
SIRPA	MPL	SH2B1	LEPR	STAM2	STAT5B
SH2B1	CSF2RB	TEC	CCR2	IL6R	STAT1
TEC	TYK2	IL23R	CTLA4	IL5RA	STAT3
IL23R	JAK1	SKB1	PRMT5	IL10RA	EPOR
SKB1	NCK1	STAM2	EZH2	IL4R	IRS1
STAM2	ROCK2	LEPR	GAB2	ITGB2	SOCS3
IFNGR2	EPOR	HSP90AB1	STAT4	IFNGR1	SOCS1
HSP90AB1	PTPN11	STRA6	STAT1	ITGAL	PTPN11
STRA6	SOCS3	RHEX	STAT3	IFNGR2	
RHEX	PTPN6	ASB2	MAP3K5	CSF2RA	
ASB2	PIK3R1	IL6ST	ARHGEF1		
	FYN	GHR	ATOH1		
	IL12RB2	STAT5B	STAT6		
	IFNGR2	STAT5A	STAT5B		
	IFNGR1	PIK3CA			

Supplementary Fig. 3 JAK-STAT3 pathway is one of nintedanib-associated signaling alterations in senescent HDFs. A KEGG pathway in nintedanib-treated senescent cells vs. control-treated senescent cells. **B and C** GSEA (**B**) of transcriptomics data obtained from nintedanib- or ABT263-treated senescent HDFs with JAK2 pathway-related genes listed in various tested databases (**C**). The normalized enrichment score (NES) and p value are indicated at the bottom of the corresponding figures.

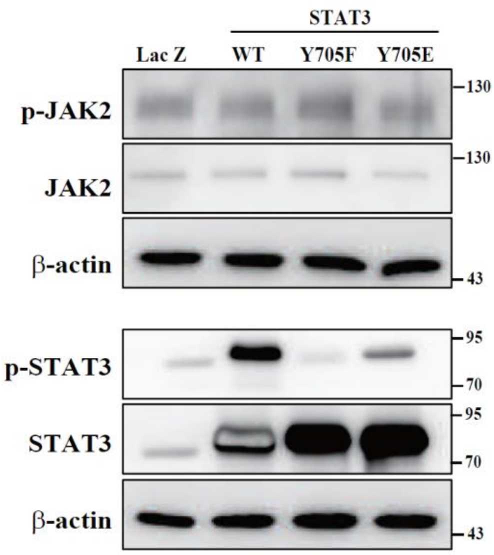

Supplementary Fig. 4 Immunoblots for validating STAT3 overexpression constructs. Nonsenescent HDFs were infected with lentiviral particles to overexpress LacZ, wild-type (WT) STAT3, or mutants STAT3 (Y705E or Y705F) proteins. Western blot assays using anti-p-JAK2, anti-JAK2, anti-p-STAT3, and anti-STAT3 antibodies were performed to determine the lentiviral transduction efficiency.

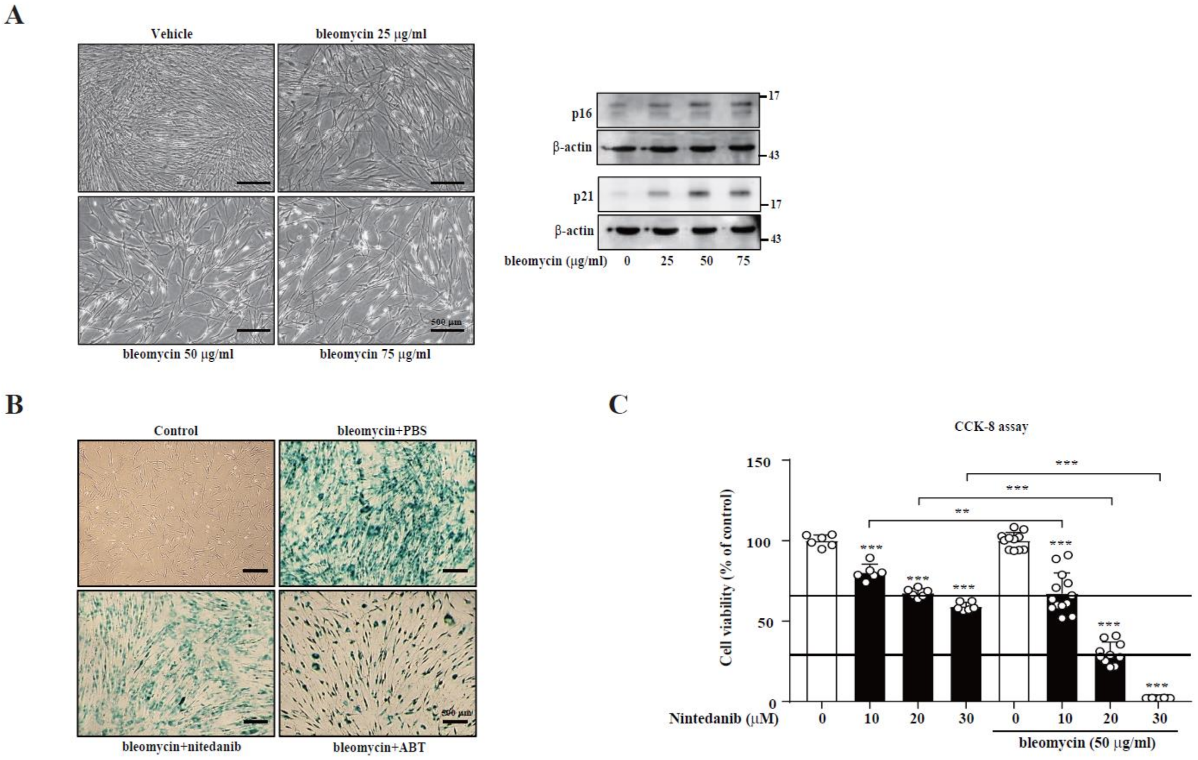

Supplementary Fig. 5 Nintedanib shows senolytic effects on bleomycin-induced cellular senescence of human pulmonary fibroblasts (HPFs). A Nonsenescent HPFs were treated with bleomycin (25, 50, and 75 μg/ml) for 5 days, and then. we confirmed cell morphological changes. Images were randomly captured (left panel). Western blot assays using anti-p16, and anti-p21 antibodies were performed to confirm bleomycin-induced cellular senescence (right panel). **B** Bleomycin-induced senescent HPFs were treated with 10 μM nintedanib, and 5 μM ABT263 for 3 days. SAβG staining was performed to measure the number of senescent cells. **C** Bleomycin-induced senescent HPFs were treated with nintedanib (10, 20, and 30 μM) for 3 days and then CCK-8 assays were performed to determine cell viability. Scale bar, 500 μm. The data normalized to those for DMSO-treated cells are shown as the mean ± S.D. *$p<0.05$, **$p<0.01$, ***$p<0.001$ by one-way ANOVA with Tukey's post hoc test.
