# Supplementary material for: Nintedanib induces senolytic effect via STAT3 inhibition
Source: Cell Death Dis. 2022 Sep 2;13(9):760. doi: 10.1038/s41419-022-05207-8 (PMC9440251; doi:10.1038/s41419-022-05207-8)

Figure 2B

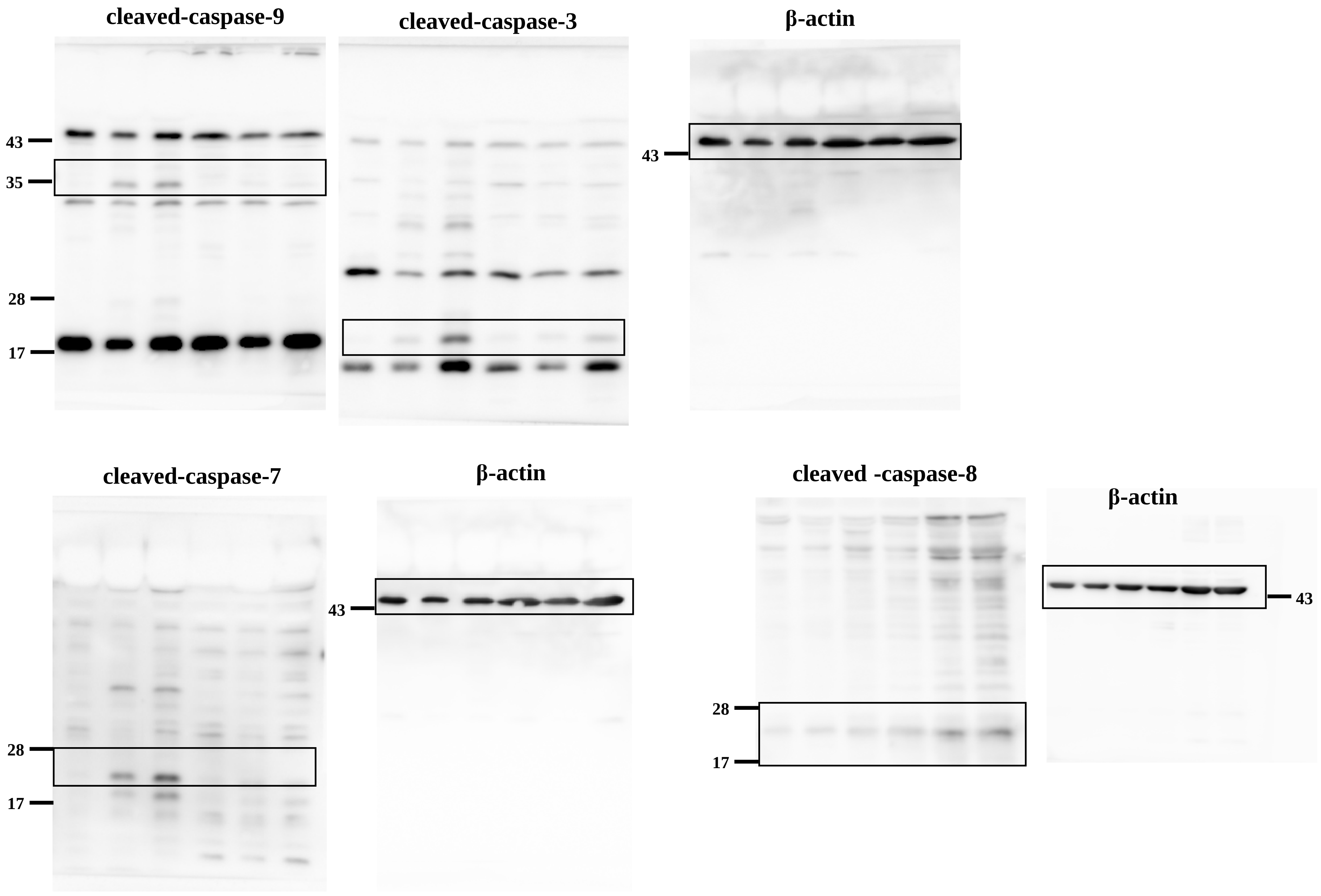
cleaved-caspase-9
cleaved-caspase-3
β-actin
43
35
28
17
43
cleaved-caspase-7
β-actin
cleaved -caspase-8
β-actin
28
17
43
28
17
43

**Figure 3C**

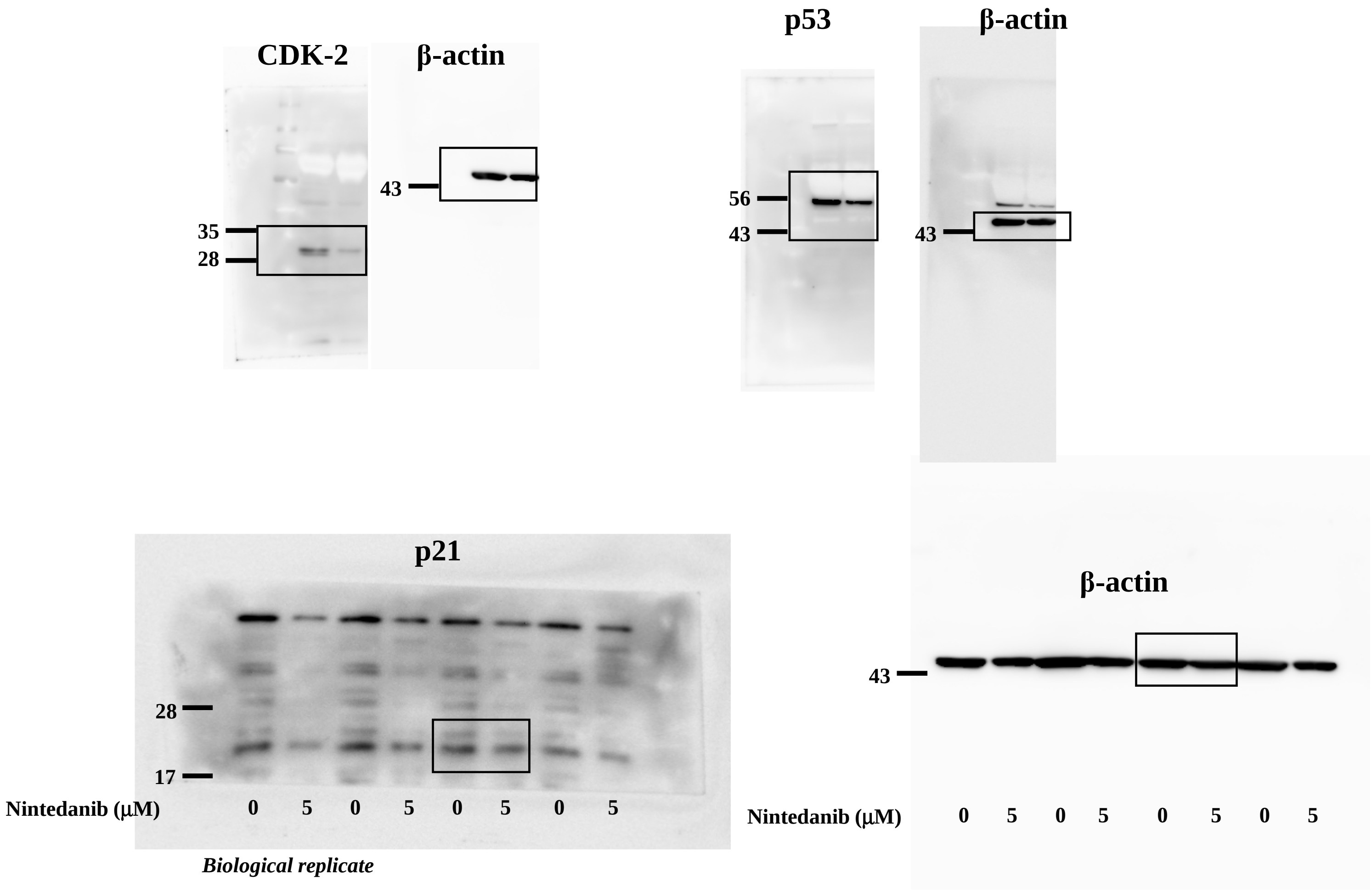
CDK-2
β-actin
35
28
43
p53
β-actin
56
43
43
p21
28
17
Nintedanib (μM) 0 5 0 5 0 5 0 5
β-actin
43
Nintedanib (μM) 0 5 0 5 0 5 0 5
Biological replicate

**Figure 3H**

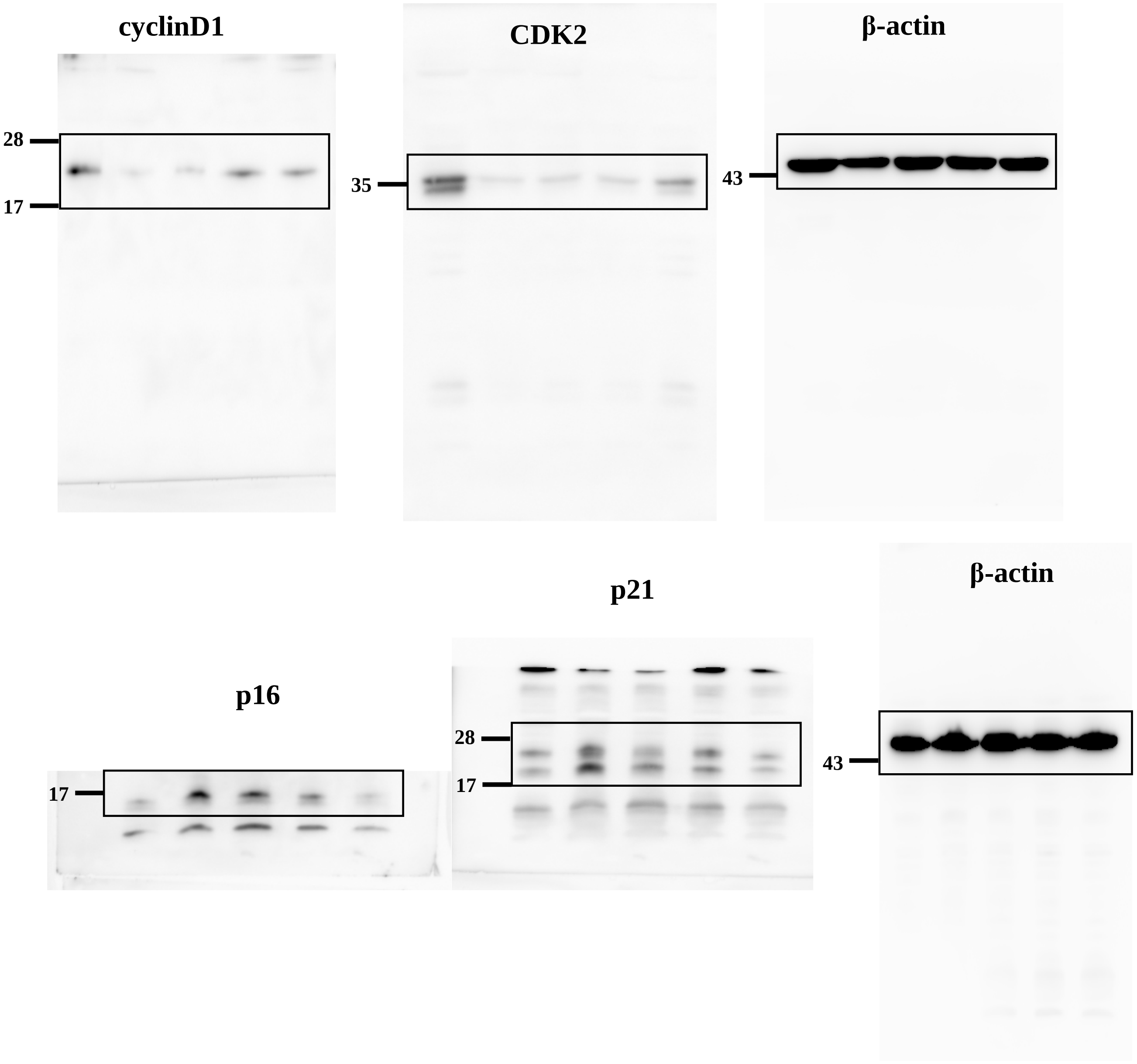

# Figure 4A

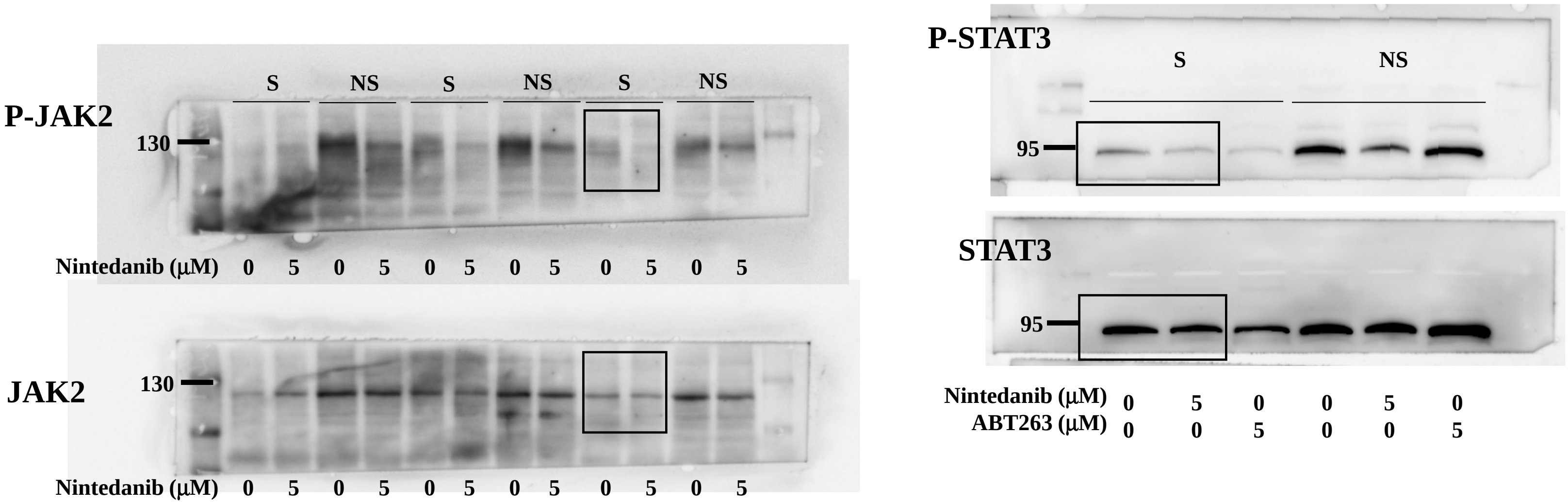


*Biological replicate*

Figure 4B

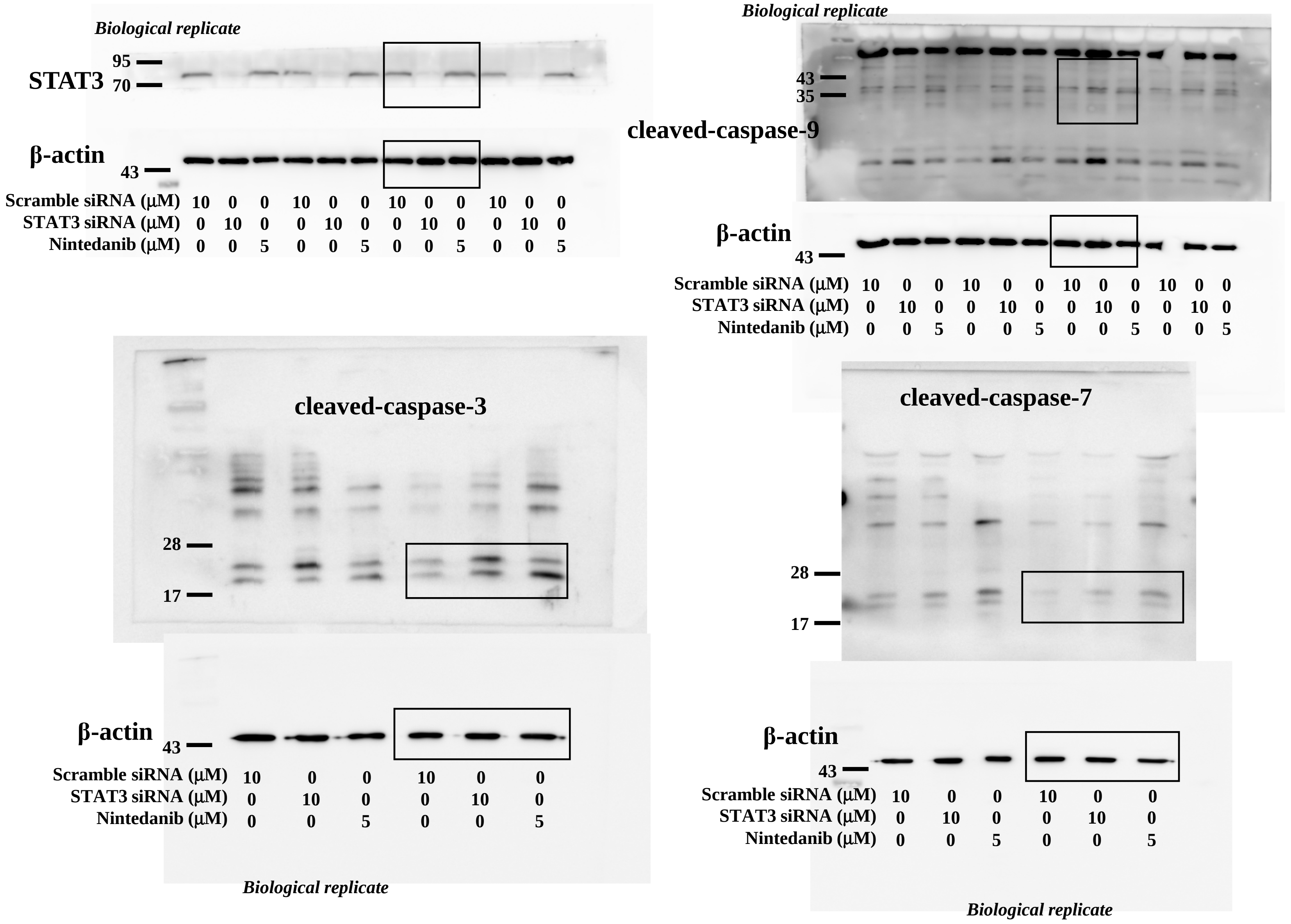
Biological replicate
STAT3
95
70
β-actin
43
Scramble siRNA (μM) 10 0 0 10 0 0 10 0 0 10 0 0
STAT3 siRNA (μM) 0 10 0 0 10 0 0 10 0 0 10 0
Nintedanib (μM) 0 0 5 0 0 5 0 0 5 0 0 5
Biological replicate
cleaved-caspase-9
43
35
β-actin
43
Scramble siRNA (μM) 10 0 0 10 0 0 10 0 0 10 0 0
STAT3 siRNA (μM) 0 10 0 0 10 0 0 10 0 0 10 0
Nintedanib (μM) 0 0 5 0 0 5 0 0 5 0 0 5
cleaved-caspase-3
28
17
β-actin
43
Scramble siRNA (μM) 10 0 0 10 0 0
STAT3 siRNA (μM) 0 10 0 0 10 0
Nintedanib (μM) 0 0 5 0 0 5
Biological replicate
cleaved-caspase-7
28
17
β-actin
43
Scramble siRNA (μM) 10 0 0 10 0 0
STAT3 siRNA (μM) 0 10 0 0 10 0
Nintedanib (μM) 0 0 5 0 0 5
Biological replicate

**Figure 5F**

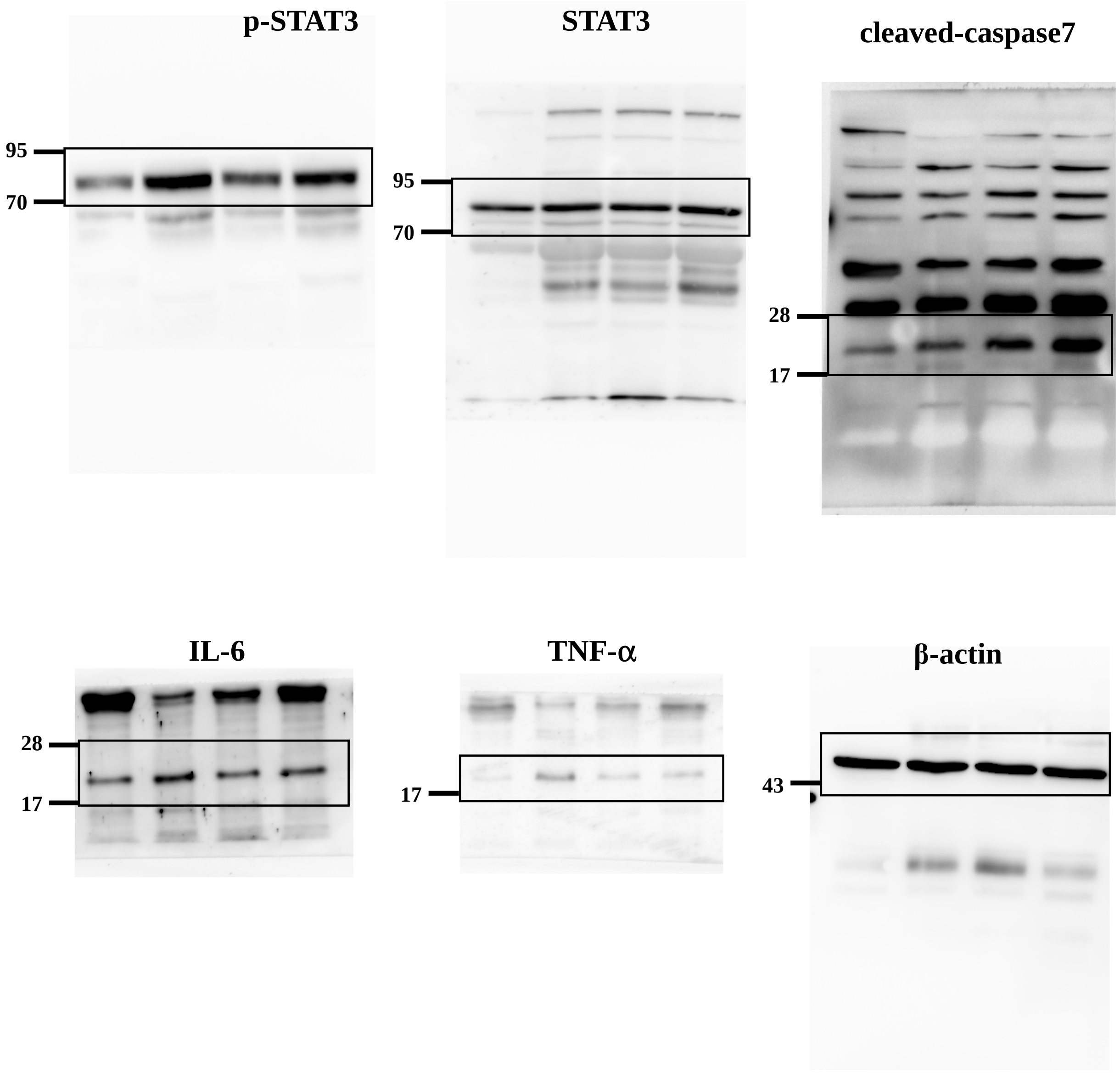
p-STAT3
95
70
STAT3
95
70
cleaved-caspase7
28
17
IL-6
28
17
TNF-α
17
β-actin
43

**Figure 5H**

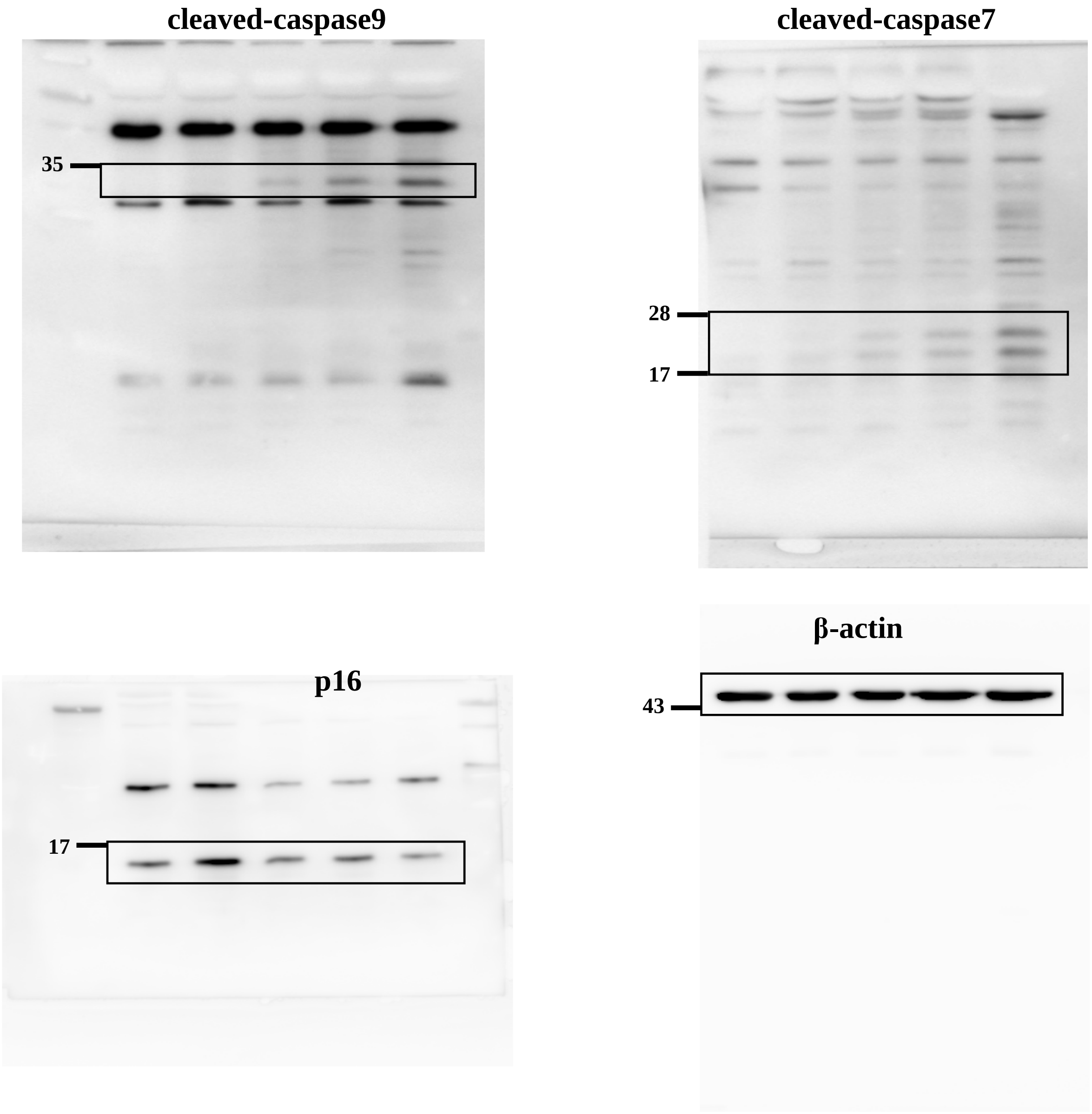

## Supplementary Figure 1

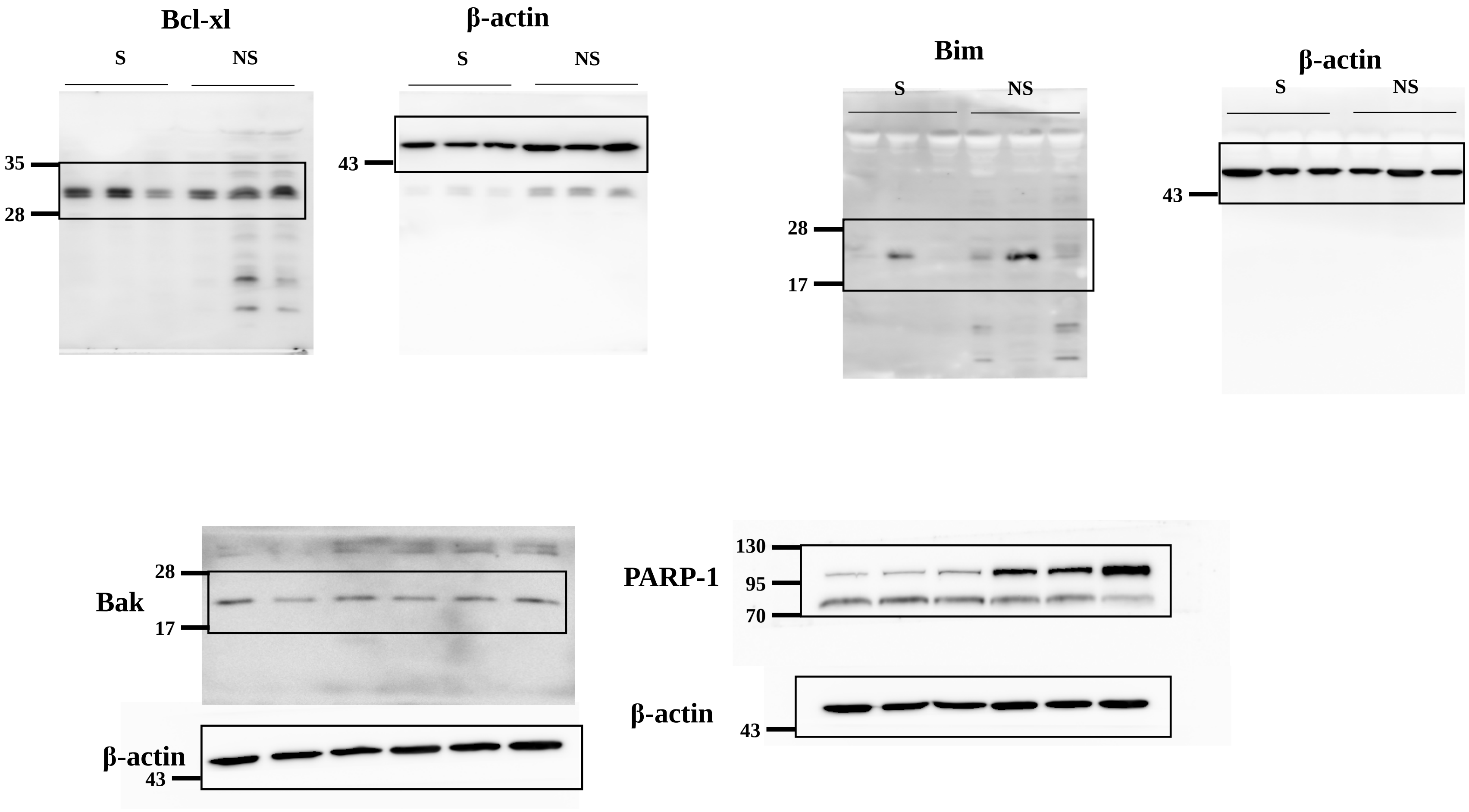

## Supplementary Figure 4A

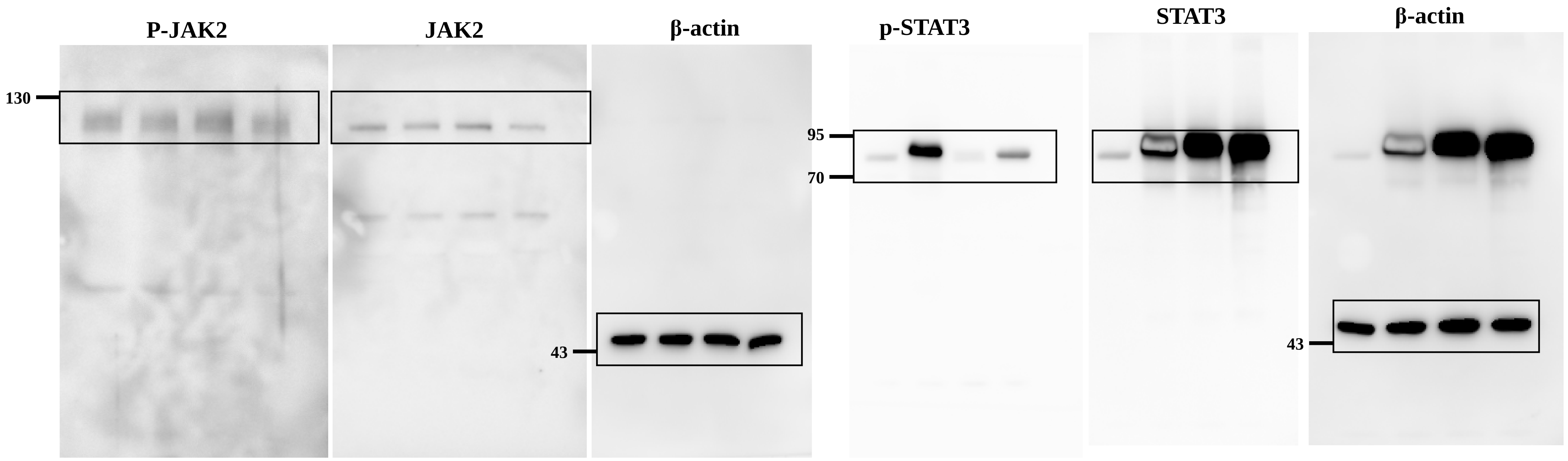

## Supplementary Figure 5A

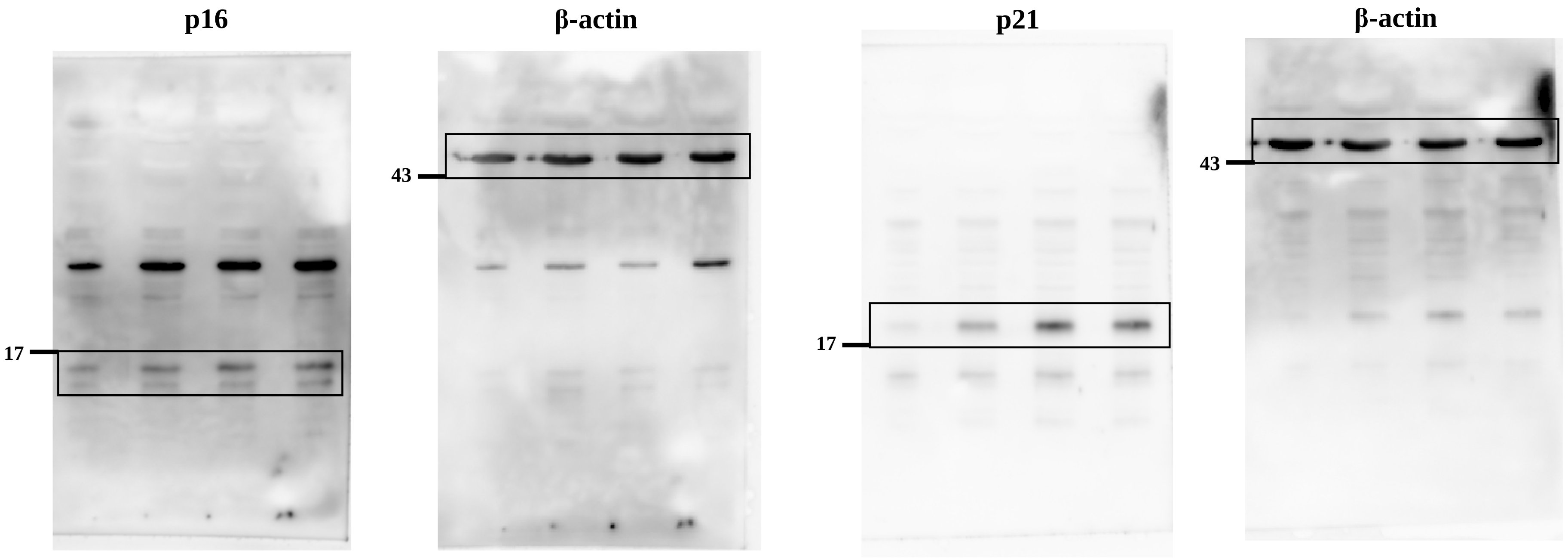

Supplement: Supplementary file 2 — Original Western Blots [file 41419_2022_5207_MOESM2_ESM.pdf]
